# Integrated Epigenetic Reprogramming of Donor Cells and Reconstructed Embryos: A Proposed Framework for Improving Bovine Somatic Cell Nuclear Transfer

**DOI:** 10.3390/biology15211985

**Published:** 2026-11-02

**Authors:** Imran Khan, Atif Ali Khan Khalil, Anukul Taweechaipaisankul, Suchinda Malaivijitnond, Jittanun Srisutush, Irene Aksoy, Pierre Savatier, Rangsun Parnpai

**Affiliations:** 1 Embryo Technology and Stem Cell Research Center, School of Biotechnology, Suranaree University of Technology, Nakhon Ratchasima 30000, Thailand; imrangnu@gmail.com; 2 Department of Biotechnology, Yeungnam University, Gyeongsan 38541, Gyeongbuk, Republic of Korea; atif.ali@yu.ac.kr; 3 Department of Biology, Faculty of Science, Chulalongkorn University, Bangkok 10330, Thailand; anukul.t@chula.ac.th (A.T.); suchinda.m@chula.ac.th (S.M.); 4 Univ Lyon, Université Lyon 1, INSERM, Stem Cell and Brain Research Institute U1208, 69500 Lyon, France; jittanunsrisutush@gmail.com (J.S.); irene.aksoy@inserm.fr (I.A.)

**Keywords:** bovine cloning, somatic cell nuclear transfer, epigenetic reprogramming, H3K9me3, DNA methylation, embryonic genome activation

## Abstract

Somatic cell nuclear transfer (SCNT) can be used to propagate valuable cattle genotypes and to support genome editing, but its efficiency is still limited by incomplete epigenetic reprogramming. The donor nucleus maintains somatic chromatin and DNA methylation patterns that may interfere with embryonic genome activation, lineage specification and development. This review highlights key barriers to reprogramming and interventions used in donor cells, reconstructed embryos, or both, and discusses the strength and species specificity of the evidence. We propose a molecularly guided two-stage framework: donor cells are primed to overcome epigenetic resistance and then assessed after washout and recovery for viability, genomic integrity and target engagement. Only when a defined molecular defect persists is a transient embryo-stage intervention considered following nuclear transfer. Candidate targets include histone H3 lysine 9 trimethylation (H3K9me3), histone acetylation, DNA methylation, and abnormal X-inactive specific transcript (XIST) expression, although some approaches require further validation in cattle. Factorial experiments should separate the effects of each intervention and their interaction. Blastocyst yield is not the only measure of success; success must also be evaluated through molecular and developmental measures including placental function, pregnancy, live birth, and postnatal health. The integrated framework is not yet validated as a complete bovine protocol but provides testable criteria for improving SCNT efficacy and safety.

## Simple Summary

Somatic cell nuclear transfer, commonly known as cloning, can be used to reproduce valuable cattle, preserve important genetics, generate gene-edited animals, and support biomedical research. However, cloning efficiency remains low because the nucleus of the donor cell is often not fully reset to an embryonic state. This review proposes a two-stage strategy in which donor cells are first prepared to become more responsive to reprogramming, and reconstructed embryos are then treated at carefully selected developmental stages to correct remaining defects. The approach focuses on targeted and temporary interventions rather than broad epigenetic changes. It also emphasizes that success should not be judged only by blastocyst formation, but also by placental development, fetal growth, calf survival, and long-term health. This integrated framework may help improve the safety, consistency, and overall effectiveness of bovine cloning.

## 1. Introduction

Somatic cell nuclear transfer (SCNT) has distinctive value in cattle because it can reproduce an otherwise irreplaceable nuclear genotype while allowing genetic modifications to be introduced and verified in cultured donor cells before embryo production [1]. The same feature that makes SCNT powerful, however, creates its central biological challenge: the oocyte receives a nucleus whose chromatin organization, transcriptional circuitry, and DNA methylation landscape were established to maintain a differentiated somatic identity [2]. Whereas a fertilized embryo begins with specialized gametic epigenomes adapted to undergo rapid remodeling, a transferred somatic nucleus contains stable heterochromatin, somatic enhancer–promoter configurations, and DNA methylation patterns that can resist the maternal reprogramming machinery [3]. The ability of the metaphase-II oocyte to remodel this information is remarkable but incomplete, and resulting defects may become apparent during cleavage, blastocyst formation, implantation, placentation, fetal growth, or the neonatal period [2,4].

The efficiency of bovine SCNT cannot therefore be adequately assessed using a single developmental endpoint. Early cleavage and blastocyst formation do not reliably predict full-term development or postnatal viability. In cattle, embryos that appear morphologically normal at the blastocyst stage may subsequently fail during gestation because of abnormal placental development and fetal loss, indicating that important epigenetic abnormalities can remain undetected during preimplantation development [5]. Similarly, in mouse SCNT models, failure to restore histone H3 lysine 27 trimethylation (H3K27me3)-dependent non-canonical imprinting causes severe placental abnormalities and post-implantation defects despite apparently normal preimplantation development to the blastocyst stage [6].

Mechanistic studies further demonstrate that aberrant DNA methylation remodeling and persistence of donor-cell epigenetic memory can disrupt lineage specification and developmental gene regulation during and after implantation [7]. Transcriptomic analyses of bovine SCNT embryos have revealed persistence of somatic gene expression programs, consistent with incomplete nuclear reprogramming and altered regulation of developmental pathways essential for embryo-to-fetus transition [3]. These observations emphasize that apparently normal preimplantation development, including normal blastocyst morphology, can conceal substantial molecular abnormalities in epigenetic reprogramming and that SCNT efficiency should ultimately be evaluated across multiple developmental stages, including gestation and postnatal development [8].

**Figure 1:**
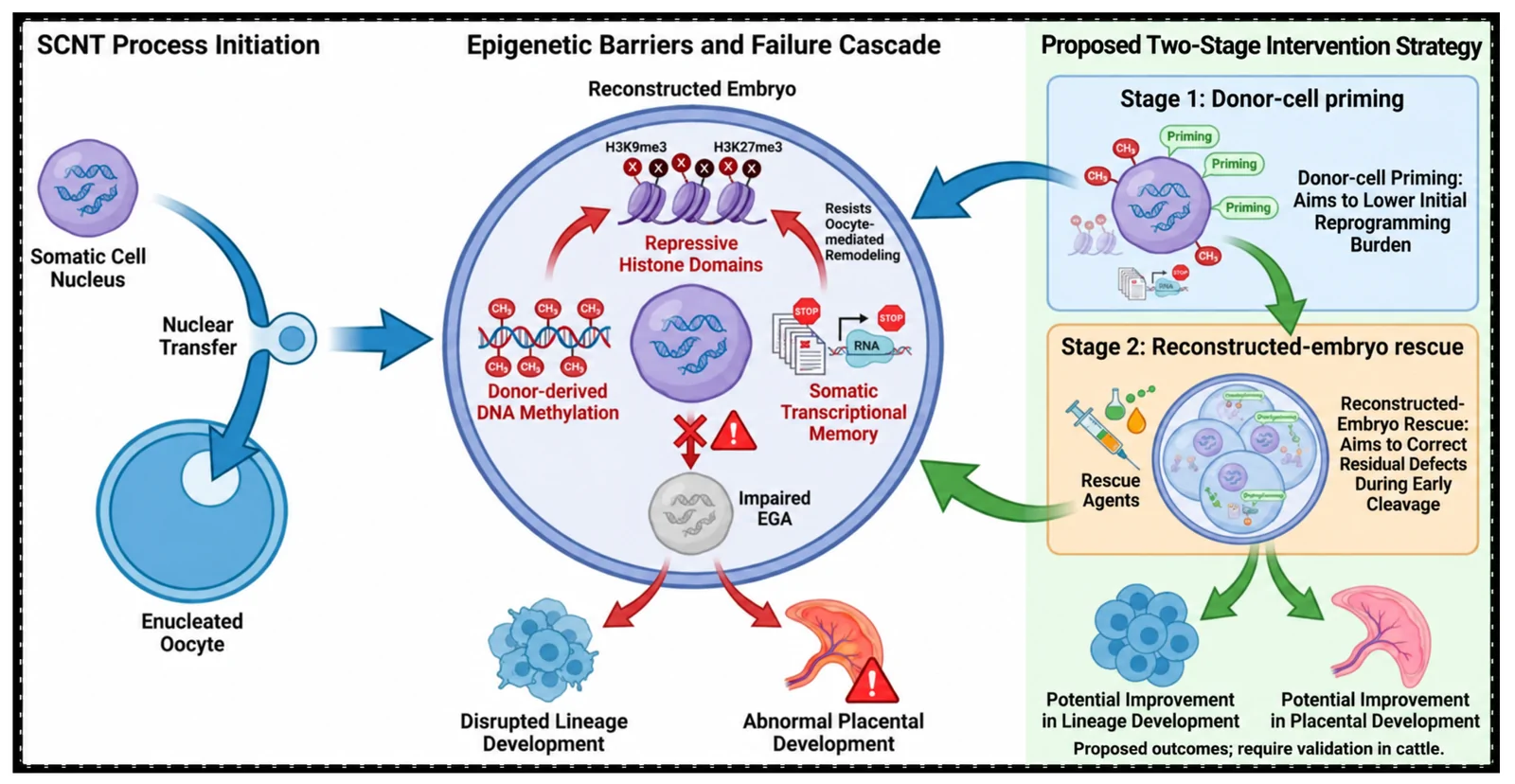
Epigenetic failure cascades in bovine somatic cell nuclear transfer and rationale for a proposed two-stage intervention. Donor-derived DNA methylation patterns, repressive chromatin domains, and somatic transcriptional memory can resist oocyte-mediated remodeling, impair embryonic genome activation, and subsequently disturb lineage specification and placental development. Donor-cell priming aims to reduce the initial epigenetic resistance of the transferred nucleus, whereas reconstructed-embryo rescue aims to correct residual reprogramming defects during early cleavage. Blue arrows indicate nuclear transfer and the proposed donor-cell intervention; red arrows, crosses, stop signs, and warning triangles indicate reprogramming barriers and potential developmental abnormalities. Green arrows indicate proposed interventions and intended developmental benefits. Potential improvements in lineage and placental development depicted on the right are hypothetical outcomes of the proposed integrated strategy and have not been experimentally established in cattle. Other colors distinguish schematic components and do not encode quantitative information. EGA, embryonic genome activation; SCNT, somatic cell nuclear transfer. Experimental studies have focused on different stages of this reprogramming process. In bovine studies, epigenetic modifiers have been applied to donor cells before nuclear transfer, to reconstruct embryos after nuclear transfer, or sequentially at both stages [13,14]. In bovine SCNT embryos, histone deacetylase (HDAC) inhibitors and KDM4 mRNA have been evaluated after nuclear transfer [9,15], whereas RNA interference-mediated Xist suppression has been investigated primarily in mouse SCNT embryos [16]. However, an epigenetic intervention that is beneficial at one stage may be ineffective or detrimental at another. Donor cells must retain viability, proliferative capacity, and genomic stability during culture, whereas reconstructed embryos require rapid and precisely timed epigenetic remodeling without disruption of DNA replication, lineage specification, or developmental progression. The objective is therefore not global hyperacetylation or hypomethylation per se, but the establishment of a developmentally permissive and reversible epigenetic state that facilitates faithful nuclear reprogramming [17].

Several molecular barriers contribute to this incomplete reprogramming. Broad histone H3 lysine 9 trimethylation (H3K9me3)-enriched domains inherited from donor chromatin can remain refractory to transcriptional activation after nuclear transfer. Transient expression of lysine demethylase 4 (KDM4)-family enzymes can partially overcome this barrier, with supporting evidence from mouse, bovine and primate SCNT studies [2,9,10]. Aberrant de novo DNA methylation can re-silence developmental genes after an initial demethylation phase: reducing this remethylation improves post-implantation development in mouse SCNT embryos. Failure to reconstruct the oocyte-specific H3K27me3 landscape can disrupt non-canonical imprinting and contribute to placental abnormalities in mouse SCNT embryos [6]. Additional defects include abnormal X-inactive specific transcript (XIST) expression, incomplete embryonic genome activation (EGA), disturbed chromatin accessibility, and persistence of somatic transcriptional memory. Importantly, these abnormalities may interact rather than operate independently. Figure 1 summarizes this multistep failure cascade and identifies potential intervention points before and after nuclear transfer [11,12].

Building on previous studies of donor-cell and embryo-stage interventions, this review proposes a molecularly guided framework combining donor-cell priming with conditional embryo-stage rescue. Donor-cell treatment is followed by washout, recovery, and defined molecular release criteria. Subsequent embryo-stage intervention is then selected according to the residual reprogramming defect rather than applied routinely. The proposed framework further disentangles the independent and interactive effects of the two treatment stages using factorial experimental designs, and evaluates success using molecular, placental, fetal, neonatal and postnatal endpoints, rather than blastocyst formation alone. Accordingly, this review aims to: (i) identify the major epigenetic barriers relevant to bovine SCNT; (ii) critically assess donor-cell and embryo-stage interventions in terms of mechanism, timing, supporting evidence, advantages and limitations; and (iii) propose testable criteria for determining whether sequential interventions provide additive or synergistic improvements in nuclear reprogramming without compromising developmental safety.

## 2. Literature Identification and Evidence Selection

This narrative review was developed using a targeted literature search for studies published online up to 2026. Relevant publications were identified using PubMed, Scopus, and Web of Science with the following search terms, alone or in combination: “bovine somatic cell nuclear transfer”, “epigenetic reprogramming”, “donor cells”, “histone modifications”, “DNA methylation”, “embryonic genome activation”, “chromatin accessibility”, and “cloning efficiency”. Original studies focusing on bovine donor cells, reconstructed embryos and developmental outcomes were given priority. Studies from other livestock species and mouse or human models were also included if they provided relevant mechanistic insights or targeted pathways that were not sufficiently studied in cattle. Evidence was evaluated by species, experimental design, molecular or developmental outcomes, and whether a causal intervention was directly tested. Findings were classified as direct bovine developmental evidence, bovine molecular or mechanistic evidence, or cross-species evidence. Particular consideration was given to studies reporting molecular reprogramming, blastocyst development, pregnancy, live birth and postnatal outcomes. As this was a narrative rather than a systematic review, formal systematic-review screening procedures and meta-analysis were not performed.

To ensure uniform interpretation, evidence was classified into four categories: (I) direct bovine developmental evidence, where experimental interventions are performed and embryonic or postnatal outcomes are measured; (II) bovine molecular or mechanistic evidence, where relevant regulatory pathways or molecular abnormalities are identified but developmental rescue is not demonstrated; (III) supporting evidence from closely related livestock species; and (IV) mechanistic or interventional evidence from mouse, human or other non-livestock models. These categories helped to distinguish between experimentally demonstrated outcomes and mechanistic associations and extrapolations across species. Importantly, evidence from one species was not considered sufficient to demonstrate the efficacy or safety of an intervention in cattle.

The conceptual figures presented in this review were generated using FigureLabs (https://chat.figurelabs.ai/ (accessed on 18 September 2026)), an AI-assisted scientific illustration platform. The tool was used to visually represent the epigenetic barriers, stage-specific interventions, and proposed integrated reprogramming framework discussed in the manuscript. These figures summarize scientific concepts and do not represent original experimental findings.

## 3. Epigenetic Barriers That Limit Bovine SCNT

The transferred somatic nucleus is not a passive DNA container but retains a three-dimensional chromatin architecture, nucleosome organization, histone-variant profile, DNA methylation landscape, and transcription factor network that collectively maintain somatic cell identity [3]. The recipient oocyte must rapidly erase these somatic epigenetic features, establish an embryonic chromatin state, and activate the embryonic developmental program within a narrow developmental window [18]. Because major EGA in cattle occurs at the 8- to 16-cell stage, defects established immediately after nuclear transfer may remain phenotypically silent during the early cleavage divisions and become apparent only at later stages, when extensive embryonic transcription is required [19].

H3K9 trimethylation (H3K9me3) is one of the most consistently identified epigenetic barriers to nuclear reprogramming. In differentiated cells, H3K9me3 is enriched in constitutive and facultative heterochromatin, transposable element-rich regions, and other repressive chromatin domains that restrict transcription factor accessibility [11,20]. In bovine SCNT embryos, insufficient lysine demethylase 4E (KDM4E) activity contributes to persistent H3K9me3 and incomplete nuclear reprogramming, whereas KDM4E overexpression promotes EGA and improves blastocyst development [9]. Similarly, studies in mouse and human SCNT systems have shown that transient expression of KDM4-family histone demethylases can overcome H3K9me3-associated reprogramming barriers and improve developmental competence [2,10]. However, excessive or prolonged depletion of H3K9 methylation may compromise heterochromatin integrity, potentially leading to repeat instability and chromosome segregation defects [21]. The objective is therefore not indiscriminate depletion of H3K9me3, but transient remodeling of donor-derived H3K9me3 barriers followed by the timely establishment of the heterochromatin landscape required for normal embryonic development [11].

DNA methylation constitutes a second major epigenetic barrier to successful nuclear reprogramming [17]. Differentiated cells retain DNA methylation patterns at specific promoters, enhancers and other regulatory regions that can restrict activation of embryonic genes, whereas methylation at repetitive elements and imprinting control regions must be selectively maintained to preserve genome integrity and genome imprinting [22]. Bovine SCNT embryos exhibit abnormal DNA demethylation and remethylation dynamics during early development, resulting in methylation patterns that differ from those of IVF embryos. In mouse SCNT embryos, suppression of excessive de novo DNA methylation through knockdown of DNMT3A and DNMT3B significantly improved post-implantation development, suggesting that aberrant locus-specific remethylation, rather than simply elevated global 5-methylcytosine levels, represents an important reprogramming barrier [23]. In bovine SCNT embryos, overexpression of ten-eleven translocation dioxygenase 3 (TET3) has been observed to enhance DNA demethylation and improve early embryonic development [24]. Consequently, indiscriminate DNA demethylation is unlikely to provide an effective reprogramming strategy, as it may disrupt genomic imprinting, activate repetitive elements, and compromise genome stability [25].

Histone acetylation is commonly used as a practical indicator of chromatin accessibility during nuclear reprogramming. SCNT embryos frequently exhibit abnormal or delayed acetylation patterns of histones H3 and H4, which may contribute to inefficient chromatin remodeling and impaired activation of embryonic transcriptional programs [26,27]. HDAC inhibitors, including trichostatin A (TSA), scriptaid, sodium butyrate, and valproic acid, have improved preimplantation development and cloning efficiency in several mammalian species by promoting histone hyperacetylation and facilitating reprogramming of the donor genome [28,29]. However, the magnitude of these effects varies with species, inhibitor concentration, exposure duration, donor-cell epigenetic state, and nuclear transfer and activation procedures [15]. Importantly, HDAC inhibitors affect multiple cellular pathways beyond histone acetylation, including acetylation of non-histone proteins, cell-cycle regulation, DNA-damage responses, and cellular metabolism [30,31]. Increased histone acetylation should therefore be interpreted as an indicator of chromatin remodeling rather than as evidence that nuclear reprogramming is complete or that the embryonic transcriptional program has been correctly reestablished.

H3K27me3 presents a more complex reprogramming challenge. In the early mammalian embryo, maternal H3K27me3 can mediate DNA-methylation-independent imprinting at a subset of genes, particularly in extraembryonic lineages [32]. A somatic nucleus does not reproduce the oocyte-specific distribution of this mark. Mouse SCNT studies have demonstrated loss of H3K27me3-dependent imprinting, biallelic activation of normally imprinted genes, and impaired placental development [4]. Because placental development and imprinting regulation differ substantially between rodents and ruminants, H3K27me3-sensitive loci identified in mouse SCNT models require direct locus-level validation before these findings can be extrapolated to bovine cloning systems. Nevertheless, the underlying principle remains relevant: broad disruption of Polycomb-mediated repression may increase accessibility at selected genomic regions while simultaneously destabilizing lineage-specific transcriptional programs or imprinting control at others [4,33].

X-chromosome regulation and genomic imprinting are additional sources of reprogramming defects. Ectopic Xist/XIST expression in cloned embryos can silence X-linked genes in inappropriate cells or developmental windows, while deficient X-chromosome dosage compensation may also be harmful [34]. Imprinted genes are important for placental and fetal development and altered expression and DNA methylation of imprinted loci have been implicated in placental abnormalities, developmental insufficiency and perinatal death in cloned cattle [35].

Abnormal EGA provides a functional link between incomplete chromatin reprogramming and developmental arrest after SCNT. Early embryogenesis is initially supported by maternally stored RNAs and proteins, which are progressively degraded and replaced by embryonic transcripts during the maternal-to-zygotic transition [36]. SCNT embryos, however, may retain donor-cell transcriptional signatures, fail to activate essential embryonic regulators, or exhibit abnormal timing and amplitude of cleavage-stage gene expression [37]. Double homeobox (DUX)-family transcription factors contribute to early embryonic transcriptional activation, although their precise functions and requirements differ among mammalian species [38]. Remodeling of chromatin accessibility and activation of regulatory elements, including repeat-derived sequences, are tightly coupled to EGA, but the underlying regulatory architecture and timing are species-specific, particularly among mice, human, and bovine embryos [39]. Single-cell transcriptomic analyses of bovine SCNT embryos have revealed abnormal transcriptional trajectories and incomplete activation of developmental programs, emphasizing that EGA markers identified in mouse embryos cannot be directly extrapolated to bovine SCNT without experimental validation [37]. Interspecies comparison of transcriptomes of SCNT embryos from five mammalian species showed that the majority of differentially expressed genes during EGA are species-specific and there are few common abnormalities. These results emphasize the importance of bovine-specific molecular targets and the need to be cautious in the direct interspecies transfer of intervention protocols [40]. Rational improvement of bovine cloning should therefore rely on the integrated assessment of species-appropriate EGA-associated gene panels and chromatin accessibility profiles rather than on individual markers derived from mouse models.

Finally, oxidative and mitochondrial stresses might worsen the failure of reprogramming in bovine SCNT embryos. Reactive oxygen species increase during micromanipulation, activation and in vitro culture and can cause mitochondrial and DNA damage but antioxidant treatment during manipulation has been reported to reduce this damage and improve nuclear reprogramming and embryo development [41]. Mitochondrial dysfunction can also reduce the supply of ATP and change the levels of redox-sensitive metabolites and cofactors needed by chromatin remodeling enzymes, thereby linking cellular stress to epigenetic regulation [42,43,44]. As physiological levels of reactive oxygen species (ROS) are involved in cellular signaling, antioxidant support should be optimized rather than used indiscriminately and should be assessed using ROS, mitochondrial membrane potential, ATP, DNA damage, apoptosis and developmental competence as complementary readouts.

The major reprogramming barriers, candidate interventions, and recommended readouts are summarized in Table 1. Together, these mechanisms illustrate why a single global modifier is unlikely to correct the full spectrum of abnormalities in SCNT embryos and why treatment strategies should instead be selected according to the specific residual molecular defects.

## 4. Reprogramming the Donor Cell Before Nuclear Transfer

**Figure 2:**
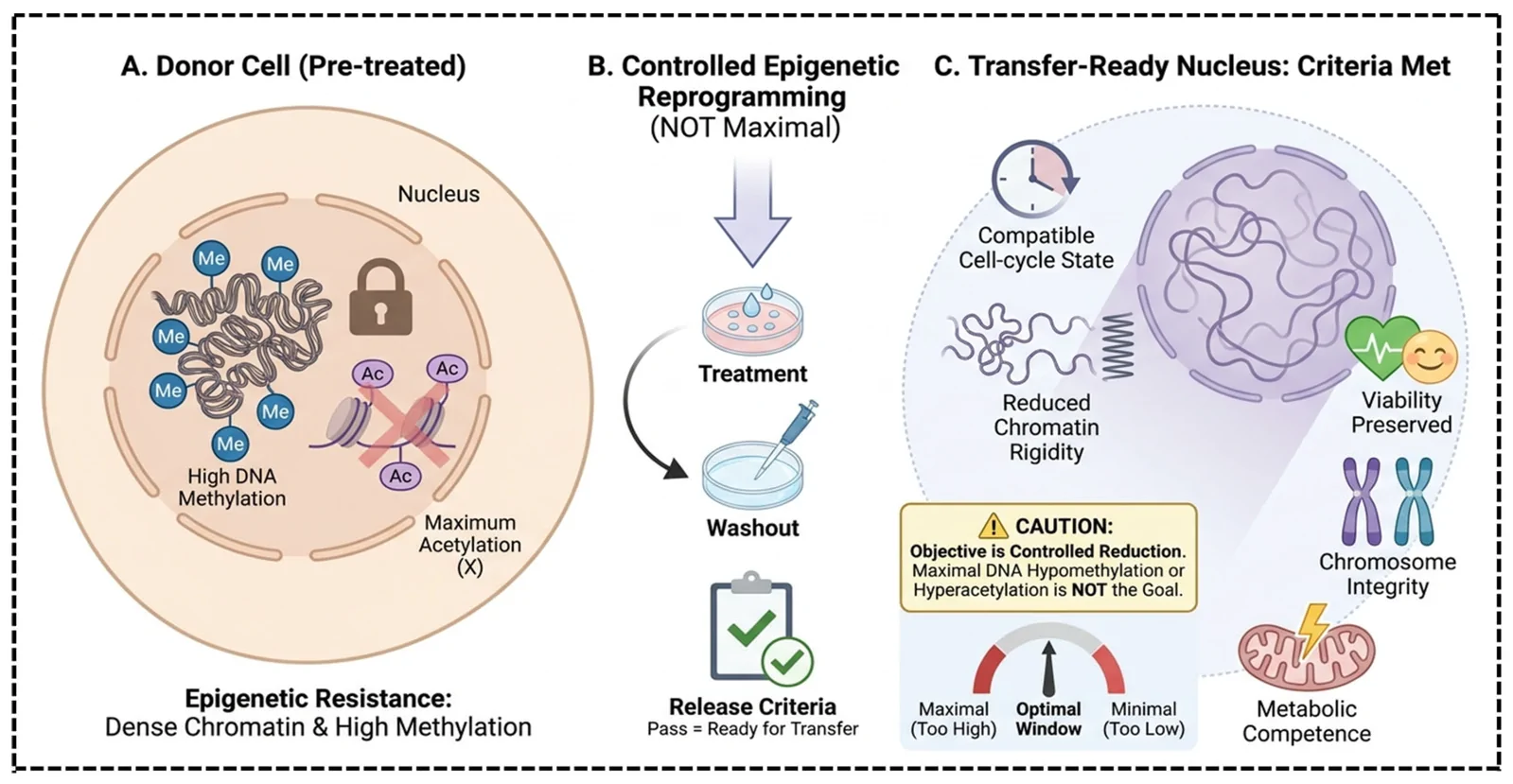
Donor-cell reprogramming as a controlled reduction of epigenetic resistance. (**A**) Schematic representation of donor-cell epigenetic resistance associated with dense chromatin and high DNA methylation. The padlock represents restricted chromatin accessibility; the red cross indicates that maximal histone acetylation is not the objective. (**B**) Proposed sequence of controlled treatment, washout, and assessment against predefined release criteria. Green check marks indicate satisfaction of these criteria rather than experimentally demonstrated developmental success. (**C**) Proposed characteristics of a transfer-ready nucleus: a compatible cell-cycle state, reduced chromatin rigidity, preserved viability, chromosome integrity, and metabolic competence. The clock represents cell-cycle compatibility; the heart and smiling face represent viability; chromosome icons represent chromosomal integrity; and the mitochondrion and lightning bolt represent metabolic competence. The gauge and warning triangle emphasize a controlled intervention window rather than maximal DNA hypomethylation or histone hyperacetylation. Colors distinguish schematic components and do not encode quantitative values. These criteria form a proposed framework requiring validation in bovine SCNT. Ac, acetylation; Me, methylation; SCNT, somatic cell nuclear transferCell source and culture history are primary determinants of SCNT efficiency. Bovine studies have demonstrated a strong effect of the donor-cell line on the efficiency and developmental outcomes of SCNT, supporting donor-cell selection as an important determinant of cloning success [49,50]. Fetal fibroblasts, adult fibroblasts, cumulus cells, and other somatic cell types differ in proliferative history, DNA methylation, chromatin compaction, and susceptibility to senescence. These differences can influence nuclear reprogramming because residual epigenetic abnormalities and incomplete chromatin remodeling may persist after nuclear transfer [46]. Low-passage cells with a normal karyotype and minimal replication stress are generally preferable, but the most readily cultured cell line is not necessarily the most suitable for cloning. Considerable differences in developmental competence have been observed among bovine donor-cell lines subjected to similar SCNT procedures [49,50]. A recent meta-analysis in bovines also linked lower passage numbers of donor cells to better cleavage and blastocyst development but these findings need to be taken with caution owing to high heterogeneity and potential bias [51]. Therefore, passage numbers should be standardized and evaluated together with the donor-cell identity, culture history and genomic integrity rather than as an independent predictor of cloning success. Reprogramming competence should therefore be regarded as a measurable cellular phenotype and assessed experimentally rather than inferred from cell availability, growth characteristics, or cell type alone. Baseline characterization should include morphology, viability, mycoplasma status, cell-cycle profile, chromosome number, DNA-damage markers, mitochondrial membrane potential, and representative epigenetic features [50].

Donor-cell treatment is an attractive strategy because cell populations can be expanded, sampled, and quality-controlled before NT. Donor-cell identity, cell-line characteristics, and residual epigenetic state influence the developmental competence of bovine SCNT embryos, emphasizing the importance of controlling donor-cell quality before nuclear transfer [49,50]. This approach also reduces the need to expose fragile reconstructed embryo to high drug concentrations. However, donor-cell interventions must meet stricter safety requirements than those typically applied in screening studies: treatments should induce a reversible increase in reprogramming competence without causing persistent DNA damage, aneuploidy, senescence, mitochondrial dysfunction, or irreversible loss of proliferative capacity. This requirement is particularly important because incomplete or excessive chromatin remodeling can compromise SCNT outcomes, and targeted epigenetic interventions that improve early embryonic development may not necessarily support long-term developmental competence [45]. Figure 2 therefore presents donor-cell reprogramming as a controlled reduction in epigenetic resistance rather than an attempt to erase the somatic epigenome completely.

Cell-cycle coordination between donor cell and recipient oocyte is essential because the transfer of a nucleus with an incompatible DNA-content state can result in ploidy abnormalities [52]. Serum starvation and contact inhibition are commonly used to enrich donor-cell population G0/G1 cells [53]. Prolonged serum starvation, however, can induce oxidative stress, apoptosis, autophagy, and extensive transcriptional changes that may compromise rather than facilitate nuclear reprogramming [54,55]. A short, empirically validated synchronization protocol, followed by an appropriate recovery period, is therefore preferable to prolonged or severe nutrient deprivation [52]. Flow-cytometric confirmation of cell-cycle distribution is important, particularly when evaluating donor-cell treatments, because treatment-induced changes in cell-cycle composition may otherwise be misinterpreted as direct epigenetic effects on reprogramming competence [53].

Transient HDAC inhibition is among the best-established epigenetic approaches for modulating donor-cell chromatin. Short-term HDAC inhibition can increase histone acetylation, reduce chromatin compaction, and potentially facilitate access of oocyte-derived reprogramming factors to the donor genome after nuclear transfer [13]. Scriptaid and TSA have been extensively evaluated in livestock SCNT systems, with studies reporting improved blastocyst development and altered expression of pluripotency-associated genes following HDAC inhibitor treatment [13,15]. However, HDAC inhibition is not restricted to chromatin remodeling. In bovine donor cells, TSA can alter the expression of cell-cycle-regulatory genes and microRNAs, emphasizing the need to optimize drug concentration, exposure duration, and washout conditions [13]. An effective donor-cell protocol should therefore identify the minimum exposure required to increase H3K9ac, H3K14ac, or H4 acetylation, while preserving cell viability, clonogenic recovery, and chromosomal integrity [56].

Reducing repressive H3K9 methylation may provide a complementary strategy to increase histone acetylation. Aberrant persistence of H3K9 methylation, particularly H3K9me3, represents a major epigenetic barrier to somatic cell nuclear reprogramming by restricting access of oocyte-derived reprogramming factors [2,9]. Chemical inhibitors targeting methyltransferases, including euchromatic histone lysine methyltransferase 2 (G9A/EHMT2) inhibitors such as BIX-01294, as well as interventions affecting suppressor of variegation 3–9 homolog (SUV39H) dependent H3K9me3, have been evaluated in donor cells or SCNT embryos in several species [9]. The conceptual advantage is the direct targeting of a well-defined reprogramming barrier. However, H3K9 methylation also contributes to heterochromatin organization, repression of repetitive sequences, and genome stability. Studies in non-bovine experimental systems indicate that extensive or prolonged disruption of H3K9 methylation can compromise these functions [21,57]. These findings should be regarded as a biologically plausible concern rather than as direct evidence of toxicity in bovine donor cells. Bovine applications therefore require careful assessment of DNA damage, chromosome integrity, repetitive-element expression, and developmental competence. Transient interventions may offer greater control than prolonged exposure of donor cells. These include inducible modulation of H3K9 methyltransferase activity or transient delivery of KDM4-family demethylases around the time of nuclear transfer [10]. In cattle, studies involving KDM4E strongly support H3K9me3 as a relevant reprogramming barrier while also emphasizing that the timing and extent of demethylation are critical determinants of developmental outcome [9].

DNA-methylation modulation has similarly produced variable outcomes. Aberrant DNA methylation remodeling is frequently observed during SCNT, with incomplete demethylation and abnormal remethylation contributing to defective embryonic gene regulation [46]. 5-Aza-2-deoxycytidine incorporates into replicating DNA and inhibits DNA methylation through covalent trapping of DNMT enzymes, whereas RG108 directly inhibits DNMT catalytic activity without incorporation into DNA. Both compounds have been investigated as potential means of reducing abnormal methylation barriers during nuclear reprogramming, although their effects depend strongly on dose, exposure duration, and cellular context [24]. Particular caution is warranted with 5-aza-2-deoxycytidine because studies in non-bovine systems have shown that DNMT trapping may stall replication forks and induce DNA damage [58]. These findings represent a general mechanistic warning rather than direct evidence of toxicity in bovine donor cells. Because treatment of donor cells necessarily involves proliferating populations, the effects of nucleoside analogues are particularly sensitive to dose, exposure duration, and proliferation history. DNA-demethylating strategies therefore require careful optimization to avoid compromising donor-cell genomic integrity and subsequent developmental competence. Global 5mC measurements alone are insufficient to assess epigenetic correction; methylation should also be evaluated at selected developmental regulatory regions, imprinted control regions, and repetitive elements [46]. Alternative approaches using vitamin C or modulation of α-ketoglutarate availability may support ten-eleven translocation (TET) and Jumonji-C dioxygenase activity through endogenous demethylation pathways, but their effects are metabolism-dependent and are not inherently locus-specific [24].

Partial reprogramming using pluripotency-associated transcription factors has been proposed as an alternative strategy for modifying the epigenetic state of donor cells [59]. Transient expressions of octamer-binding transcription factor 4 (OCT4), SRY-box transcription factor 2 (SOX2), Krüppel-like factor 4 (KLF4), MYC proto-oncogene (MYC), NANOG homeobox (NANOG), or related factors can attenuate aspects of somatic identity without inducing complete conversion to pluripotency [60]. In principle, such partial reprogramming could increase the plasticity of the donor nucleus and facilitate subsequent reprogramming by the oocyte [61]. In practice, however, heterogeneous cell-state transitions, incomplete reversal after factor withdrawal, and oncogenic or proliferative stress represent important concerns [62]. For application to bovine cloning, a transient, non-integrating expression system combined with clearly defined molecular release criteria would therefore be required. Increased expression of pluripotency-associated markers alone should not be considered evidence that donor cells are better suited for SCNT. The relevant endpoint is improved embryonic reprogramming and developmental competence while preserving donor-cell genomic integrity and proliferative capacity [63].

Direct bovine evidence does not always support pluripotency factor-mediated donor-cell priming. Another study showed that exogenous expression of OCT4 or SOX2 in bovine fibroblasts did not significantly enhance fusion, cleavage or blastocyst development after SCNT compared to control donor cells [63]. These findings suggest that increased expression of pluripotency-associated factors is not always beneficial for nuclear reprogramming competence. While this study did not directly evaluate transient priming followed by factor withdrawal and recovery, it questions the assumption that merely expressing pluripotency factors enhances donor-cell suitability. Therefore, transcription factor-induced priming remains an experimental approach that needs to be directly validated for epigenetic modifications, cell recovery, and subsequent embryonic and postnatal outcomes in cattle.

Metabolism is an underexplored component of donor-cell preparation. Several metabolic intermediates act as substrates or cofactors for chromatin-modifying enzymes, thereby linking cellular metabolism to the epigenetic state of donor cells [42]. Acetyl-CoA availability influences histone acetylation, S-adenosylmethionine provides methyl groups for DNA and histone methylation, NAD+ regulates sirtuin activity, and α-ketoglutarate serves as a cofactor for TET and Jumonji demethylases [24]. Oxidative stress must also be considered because excessive reactive oxygen species can induce damaged DNA and alter the activity of chromatin-regulating enzymes [54]. In bovine systems, donor-cell culture parameters, including oxygen tension and serum concentration, can influence subsequent SCNT outcomes, although optimal conditions may vary among donor-cell lines [64]. Conversely, excessive antioxidant supplementation may perturb physiological redox homeostasis. Metabolic interventions should therefore be empirically optimized and accompanied by assessment of mitochondrial function, ROS levels, ATP production, and apoptosis [41].

A practical donor-cell protocol should include a washout and recovery phase followed by a defined quality-control decision before nuclear transfer. Recovery after epigenetic or cell-cycle manipulation is important because transient treatments can induce cellular stress responses that persist beyond the exposure period and affect subsequent reprogramming competence [54]. We suggest that only donor-cell populations displaying high viability, an appropriate G0/G1 fraction, preserved chromosome integrity, low levels of DNA damage, stable mitochondrial function, and evidence of the intended epigenetic change should be used for SCNT. Treated cells should then be compared with untreated controls in a pilot SCNT assay before proceeding to large-scale embryo production. This framework avoids selecting a donor-cell treatment solely because it modifies an epigenetic marker, without demonstrating a corresponding improvement in embryonic developmental competence.

## 5. Reprogramming Reconstructed Embryos After Nuclear Transfer

The second major window for intervention is the reconstructed embryo. After nuclear transfer, the somatic genome is extensively re-programmed by oocyte factors, and the efficiency of this process influences subsequent embryonic development [2]. The donor nucleus is exposed to maternal histones, histone chaperones, chromatin-remodeling complexes, maternal RNAs and metabolic substrates that collectively regulate chromatin accessibility, transcriptional activation and the establishment of the embryonic epigenetic state [24].

The quality of oocytes, an important factor of the competence for nuclear reprogramming, should also be considered, in combination with the epigenetic state of donor cells [65]. The recipient oocyte provides maternal RNAs, proteins, histone chaperones and chromatin-remodeling machinery, which help in remodeling the transferred nucleus and in establishing the embryonic developmental program [17]. The development of bovine SCNT is dependent on successful oocyte maturation, appropriate nuclear transfer, activation and embryo culture conditions. Differences in the source and conditions of oocyte maturation could therefore contribute to variation in developmental competence, even when the same donor-cell line is used [65]. Donor-cell priming and embryo-stage epigenetic interventions should be a supplement, not a substitute, of sufficient oocyte competence. Therefore, in an effort to tease apart the effects of epigenetic interventions from those of recipient oocyte competence, experimental validation of the suggested framework must take into consideration oocyte origin, maturation conditions and oocyte-batch variance.

The main advantage of intervening at the embryo stage is that treatment can have a direct effect when somatic chromatin is being remodeled and the embryonic program is set up. However, this developmental window is particularly sensitive and excessive manipulation during the early cleavage stages may disrupt normal developmental progression and epigenetic resetting [46]. Thus, the therapeutic window is narrow: prolonged or nonspecific treatment may interfere with DNA replication, chromosome segregation, cleavage, imprint maintenance and lineage allocation [15]. Table 2 summarizes representative embryo-stage interventions, distinguishing direct bovine evidence from bovine mechanistic findings and evidence from other species.

Transient delivery of KDM4-family histone demethylases has provided some of the strongest mechanistic evidence for targeted epigenetic correction after nuclear transfer. In buffalo SCNT embryos, injection of KDM4D mRNA improved developmental competence, supporting the concept that transient removal of H3K9me3 can facilitate embryonic reprogramming [66]. In cattle, studies have further demonstrated that insufficient KDM4D/KDM4E activity contributes to persistent H3K9me3 barriers and defective embryonic genome activation, whereas KDM4-mediated correction of abnormal H3K9 methylation improves transcriptional activation and blastocyst development [9]. A major advantage of mRNA delivery is its temporal nature: the demethylase is rapidly expressed and subsequently degraded, allowing transient epigenetic remodeling without permanent genomic modification [66]. Dose and timing must nevertheless be carefully controlled, as excessive or prolonged H3K9 demethylation may compromise heterochromatin organization and developmental stability [45]. Accordingly, treatment efficacy should be assessed not only by blastocyst development but also by H3K9me3 reduction and restoration of embryonic transcriptional programs [9].

Combined inhibition of histone methylation pathways has also been investigated in recent bovine SCNT studies. Treatment with RK-701 and GSK-126 has been shown to decrease H3K9me3 and H3K27me3, respectively, with consequent improvements in EGA and blastocyst development [67]. However, the best treatment conditions and long-term developmental safety are yet to be determined. These results support further investigation of embryo-stage epigenetic modulation but do not show superiority of sequential donor-cell priming and embryo-stage rescue.

Embryo exposure to HDAC inhibitors remains a widely used strategy in SCNT. Recent studies continue to show that transient HDAC inhibition increases histone acetylation and can facilitate donor-chromatin remodeling during early embryonic development [68]. TSA, scriptaid, sodium butyrate, valproic acid, and related compounds have been reported to increase histone acetylation, promote nuclear remodeling, chromatin accessibility, and expression of developmental genes, with associated improvements in preimplantation development [69]. Scriptaid is often considered less toxic than TSA, but no HDAC inhibitor is universally optimal. Developmental responses vary among compounds because individual inhibitors differ in HDAC selectivity, potency, and effects on embryonic transcriptional programs [68]. Timing is also critical, particularly in relation to fusion, activation, and the first round of DNA replication. A short post-activation exposure may facilitate remodeling, whereas prolonged treatment extending into later cleavage stages may interfere with the histone deacetylation required for lineage segregation [70]. Because HDAC inhibitors also affect numerous non-histone substrates, morphological assessment of embryos should be complemented by measurements of apoptosis, chromosome integrity, and transcriptional reprogramming [71]. More selective modulation of class I HDAC activity may be a useful direction for future experiments. However, inhibition of HDAC1 and HDAC2 should be approached with caution, as their lysine-deacetylase activity is required for appropriate histone-acetylation reprogramming, chromatin regulation, and zygotic genome activation in mouse and bovine embryos [72,73].

DNA methyltransferase inhibition after nuclear transfer may reduce aberrant remethylation of the donor genome. Recent studies indicate that modulation of DNA methylation pathways can improve nuclear reprogramming by correcting abnormal methylation dynamics established after SCNT [74]. In mouse SCNT embryos, excessive de novo DNA methylation has been identified as a causal barrier to post-implantation development, and reducing DNMT3A/3B-dependent remethylation improves developmental competence [23]. In bovine SCNT embryos, treatment with DNMT inhibitors or supplementation with metabolites that support active DNA demethylation has, in some studies, increased blastocyst development and altered the expression of pluripotency-associated and imprinted genes [14]. However, indiscriminate demethylation carries substantial risks because early embryos must preserve methylation at imprinting control regions and repetitive elements [75]. A rational intervention should therefore use the lowest effective dose and a restricted exposure window. Locus-specific analyses should be carried out to confirm correction of aberrant methylation at relevant developmental regions without widespread imprint erasure [74]. Bovine-specific data further support the significance of maintaining DNA methylation at imprinted loci during nuclear reprogramming. It has been shown that overexpression of ZFP57 in bovine donor fibroblasts reduced aberrant hypomethylation of selected imprinting control regions, including H19/IGF2, and improved SCNT blastocyst development and cellular quality [76]. The findings suggest that ZFP57-mediated imprint maintenance may be a donor-cell intervention and support locus-specific methylation assessment versus global demethylation alone. Therefore, H19/IGF2 and other experimentally validated bovine imprinted regions could be utilized as molecular readouts with methylation patterns compared to stage-matched IVF controls. However, improved preimplantation development does not necessarily establish normal imprint regulation during gestation or postnatal development. The longer-term safety of this approach remains to be determined.

H3K27me3-dependent non-canonical imprinting requires a different intervention logic. Unlike H3K9me3-associated reprogramming barriers, which can potentially be alleviated by transient demethylation, H3K27me3-dependent imprinting relies on precisely established maternal chromatin states and allele-specific regulation [77]. Because the transferred somatic nucleus lacks the oocyte-specific H3K27me3 landscape, simply inhibiting enhancer of zeste homolog 2 (EZH2) or broadly reducing H3K27me3 is unlikely to recreate the appropriate maternal epigenetic state. SCNT-associated abnormalities in H3K27me3-dependent imprinted genes therefore reflect incomplete epigenetic reconstruction of allele-specific chromatin states rather than a simple excess or deficiency of Polycomb activity [78]. Mouse studies indicate that loss of H3K27me3-dependent imprinting is an intrinsic feature of SCNT that contributes to abnormal placental development [4]. Potential corrective strategies could instead include targeted repression of aberrantly expressed non-canonical imprinted genes, trophoblast-specific corrections, or engineering of donor cells to approximate the required allelic-specific state [79]. These approaches remain experimental, particularly in cattle, and H3K27me3-dependent imprinting should therefore not be approached simply through routine pharmacological inhibition of Polycomb activity.

Abnormal XIST expression represents an attractive target because ectopic X-chromosome silencing has been associated with cloning defects in mouse and primate embryos [80]. Potential approaches for modulating XIST expression include siRNA, antisense oligonucleotides, CRISPR interference, and donor-cell genome editing; however, their effectiveness and developmental safety for correcting XIST-associated abnormalities in bovine SCNT remain insufficiently established [81]. However, sex, developmental stage, and species-specific mechanisms of X-chromosome regulation must be considered [82]. Complete or prolonged XIST suppression may be beneficial during an early window of aberrant expression but detrimental later, when appropriate dosage compensation becomes necessary [83]. For bovine SCNT embryos, a transient early intervention, combined with sex-stratified analysis, is therefore more defensible than permanent XIST disruption (knockout), unless its long-term developmental safety has been established [84].

Bovine-specific observations of development provide an important reference for assessing X-chromosome regulation. Conversely, nuclear accumulation of XIST RNA starts at the morula stage in female bovine embryos, but colocalization with H3K27me3 is only visible at the day 7 blastocyst stage [85]. These findings suggest that XIST expression and chromatin-associated XCI markers have different developmental dynamics and emphasize the significance of considering embryo sex and developmental stage when investigating aberrant XIST regulation. These results were obtained from normally developing bovine embryos, and do not prove that aberrant XIST expression is a causal barrier to bovine SCNT, or that its suppression improves cloning success. Therefore, XIST-directed intervention must still be considered an experimental approach requiring direct validation in sex-stratified bovine SCNT embryos, and evidence for developmental rescue by Xist suppression is based mainly on cloning studies in the mouse [16].

Rescue of EGA is an important goal in bovine SCNT as incomplete nuclear reprogramming may interfere with the transition from somatic to embryonic transcriptional regulation [3]. Unlike the mouse two-cell model, the major bovine EGA occurs predominantly during the 8- to 16-cell stage, with transcriptional activation beginning earlier [39]. Thus, a bovine-specific assessment panel could be composed of stage-dependent expressions of ZSCAN4, KLF4 and selected EGA-associated transcriptional regulators, in combination with wider measures of embryonic transcriptional activation and repression of donor-cell gene expression. Transcriptomic analyses of bovine embryos support stage-resolved transcriptional signatures rather than individual markers [86]. Significantly, ZSCAN4 depletion impairs bovine preimplantation development and alters ZGA-associated gene expression [87]; however, its requirement during normal embryogenesis does not mean that ZSCAN4 overexpression would improve reprogramming or development of SCNT embryos [87]. Thus, candidate EGA markers should be tested against stage-matched IVF controls at the 4-, 8- and 16-cell stages and validated for their expression patterns and predictive value in bovine SCNT before being used to guide embryo-stage interventions.

Additional embryo-stage interventions include modulation of apoptosis and autophagy, antioxidant supplementation, mitochondrial support, and optimization of oocyte activation. Mitochondrial activity and cellular stress responses are important determinants of SCNT embryo competence, as impaired redox homeostasis and mitochondrial dysfunction can contribute to developmental failure after nuclear transfer [43]. Although these interventions are not strictly epigenetic, they can influence chromatin remodeling through metabolic and signaling pathways. Metabolites such as acetyl-CoA, NAD+, and α-ketoglutarate regulate chromatin-modifying enzymes and thereby connect embryonic metabolic state with epigenetic remodeling [42]. Redox state can influence TET and Jumonji-C dioxygenase activity; ATP is required for ATP-dependent chromatin-remodeling complexes; and activation-induced calcium dynamics regulate cell-cycle progression [44]. Epigenetic rescue should therefore complement, rather than substitute for, competent oocyte maturation, minimally disruptive enucleation, efficient fusion and activation, and physiological embryo culture [65].

The most informative embryo-stage experiments should use a time-resolved design. SCNT embryos undergo dynamic and incomplete molecular reprogramming, making assessments across multiple developmental windows more informative than analysis at a single endpoint [3]. Before major EGA, embryos should be evaluated for nuclear remodeling, H3K9me3 and H3K27me3 dynamics, histone acetylation, 5mC/5hmC and DNA damage. During major EGA, analysis should focus on the activation of bovine-specific transcriptional programs and chromatin accessibility. At the blastocyst stage, lineage allocation, apoptosis, imprinting, and chromosome integrity should be assessed. Embryo transfer should proceed only after a protocol has demonstrated coherent molecular and developmental improvement rather than an increase in blastocyst formation or morphological quality alone [45].

**Table 2:** Species-specific evidence and treatment parameters for embryo-stage interventions in SCNT.

Intervention	Evidence	Delivery	Start/Stop Window	Dose/Duration	Principal Finding	Ref.
KDM4E mRNA	I	mRNA injection	6 h post-activation	10 pL; 800 ng/µL	↓ H3K9me3; ↑ EGA/development	[9]
TSA/scriptaid	I	Culture medium	Post-activation; 20 h	TSA: 5 nM; SCR: 5–500 nM	↑ H4K12ac; ↑ blastocysts	[15]
SAH	I	Culture medium	Post-fusion; tested 12–72 h	1 mM; 12 h (yield), 48 h (5mC)	Altered 5mC; ↑ development	[47]
Xist RNAi	IV	RNA interference	Early mouse embryo; bovine untested	Bovine dose/window unknown	Improved X-linked regulation (mouse)	[16]
KDM5B mRNA	II	mRNA injection	Reconstructed embryo	Not specified here	↓ somatic memory; ↑ developmental genes	[48]
Melatonin	I	Culture medium	During embryo culture	Not specified here	↓ ROS/apoptosis; ↑ quality	[88]

**Notes:** Evidence categories: I, direct bovine developmental evidence; II, bovine molecular or mechanistic evidence; III, evidence from closely related livestock species; IV, mechanistic or interventional evidence from mouse, human, or other non-livestock models. These categories describe the nature of supporting evidence and do not necessarily establish the efficacy, reproducibility, or developmental safety of the interventions in bovine SCNT. Experimental parameters not established from the cited studies are identified accordingly. Treatment conditions are study-specific; doses and exposure periods are reported only where supported by the cited studies. Unverified parameters are identified accordingly. **Abbreviations:** NT, nuclear transfer; EGA, embryonic genome activation; SAH, S-adenosylhomocysteine; SCR, scriptaid; TSA, trichostatin A; ROS, reactive oxygen species; ↑, increased; ↓, decreased.

## 6. Integrated Donor-Cell and Embryo Reprogramming: A Proposed Experimental Framework

Previous studies have used epigenetic interventions on bovine donor cells, reconstructed embryos, or both. Cloned calves have been produced by treating both donor cells and embryo stages [14], showing feasibility but not identifying which stage-specific defects were corrected or whether the treatments were independent or interactive. The proposed framework therefore restricts embryo-stage rescue to molecular defects remaining after donor-cell priming. This is not a validated bovine production protocol, but a testable experimental strategy.

The central premise of integrated reprogramming is that donor-cell and embryo-stage interventions can address different components of the same reprogramming burden. Donor-cell priming aims to reduce the resistance of the incoming somatic epigenome by modifying chromatin organization, cellular state, and transcriptional memory before nuclear transfer [55]. In contrast, embryo-stage rescue acts during oocyte-driven remodeling to correct residual defects in chromatin remodeling, transcriptional activation, and embryonic developmental programming after nuclear transfer [33,39]. Applying the same compound at both stages does not therefore constitute an integrated strategy by itself. Integration should involve complementary mechanisms, limited and preferably non-overlapping toxicities, and molecular evidence that the second intervention corrects a defect that persists after the first [71].

A useful conceptual model is that of a reprogramming threshold. Each donor-cell line can be considered to have an initial level of reprogramming resistance determined by features such as H3K9me3-enriched domains, DNA methylation, somatic transcriptional memory, senescence, and chromatin architecture [46,55]. The recipient oocyte provides maternal factors and cytoplasmic components essential for nuclear remodeling and subsequent development following nuclear transfer [65]. Within the proposed framework, donor-cell priming is intended to reduce initial reprogramming resistance, whereas embryo-stage rescue is designed to address residual molecular defects that persist after nuclear transfer. Successful development would require sufficient reprogramming before and during major EGA while preserving appropriate imprinting and lineage specification [89]. This model could explain why an intervention benefits a relatively resistant donor-cell line but has little effect on a more permissive line, or why an embryo-stage treatment is ineffective when donor-cell damage is the dominant problem.

**Figure 3:**
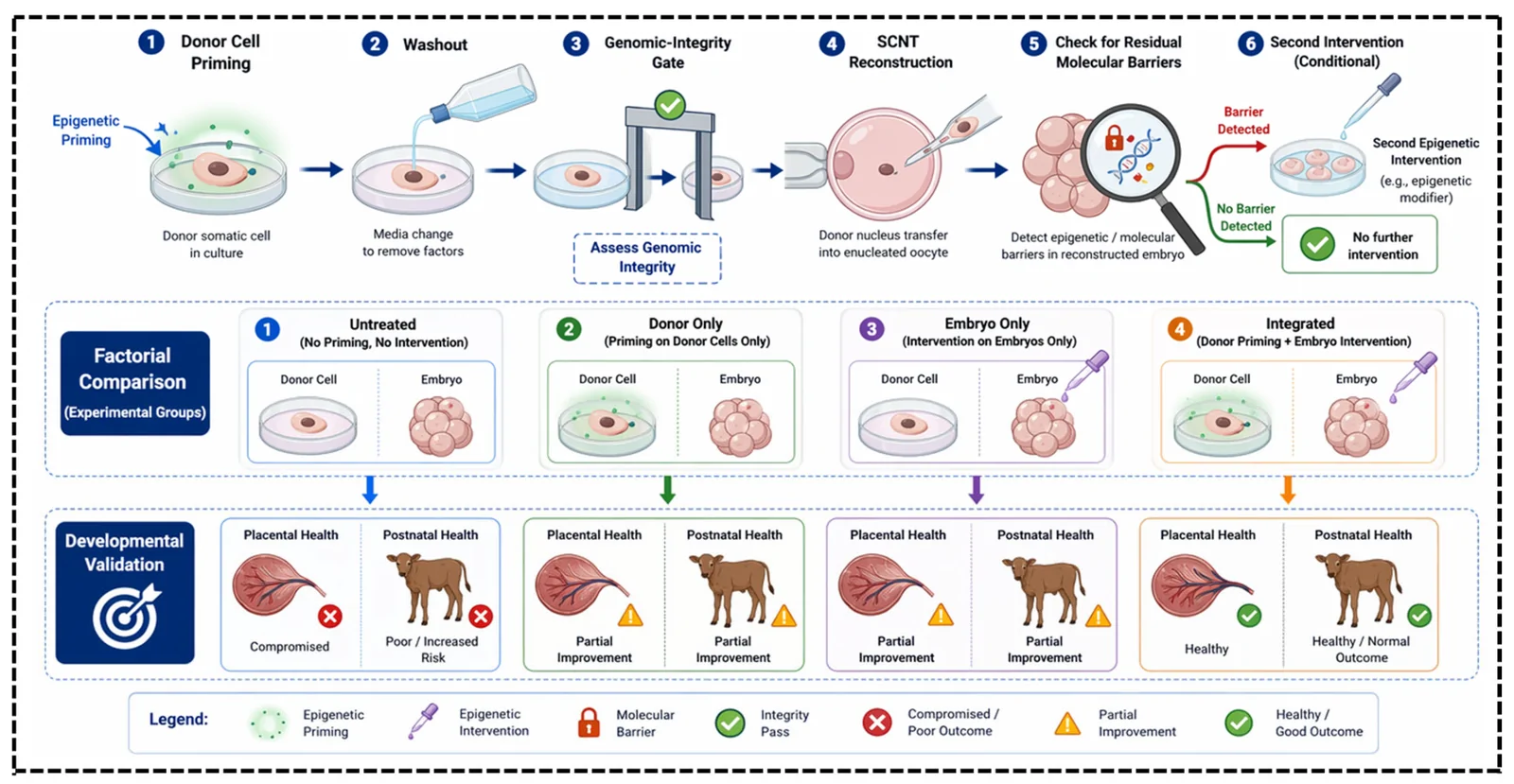
Integrated donor-cell priming and conditional embryo-stage intervention framework for bovine SCNT. The proposed workflow comprises donor-cell priming, washout and recovery, genomic-integrity assessment, SCNT reconstruction, assessment of residual molecular barriers, and conditional embryo-stage intervention. Molecular assessment initially relies on parallel pilot cohorts rather than destructive testing of individual embryos intended for transfer. Blue arrows indicate workflow progression; the red branch indicates detection of a residual barrier, whereas the green branch indicates no further intervention. Green dots represent donor-cell priming, pipettes represent embryo-stage intervention, and padlocks represent molecular barriers. Blue, green, purple, and orange distinguish the untreated, donor-only, embryo-only, and integrated experimental groups, respectively. Check marks at the genomic-integrity gate indicate satisfaction of proposed release criteria. In the developmental-validation row, red crosses, warning triangles, and green check marks depict hypothetical adverse, intermediate, and favorable outcomes, respectively; these are illustrative scenarios, not established treatment effects or guaranteed outcomes. All four groups require comparative evaluation of placental, neonatal, and postnatal outcomes. SCNT, somatic cell nuclear transfer.

The proposed workflow begins with baseline profiling (Figure 3). Candidate donor-cell lines should be characterized using markers relevant to reprogramming competence rather than selected solely on the basis of proliferation capacity. A proposed baseline panel could include cell-cycle distribution, karyotype, phosphorylated histone H2AX (γH2AX), mitochondrial function, global H3K9me3, expression of representative genes located within H3K9me3-resistant domains, total 5mC and methylation at selected developmental and imprinted loci [90]. Single-cell RNA sequencing is valuable for identifying transcriptional heterogeneity during bovine preimplantation development [91]. Based on these discovery level data and mechanistic literature outlined above, a targeted qPCR panel could be developed for the screening of donor cells. However, the predictive value of this proposed donor-cell biomarker panel for bovine SCNT outcomes remains to be established through prospective validation.

Stage 1 consists of a short donor-cell intervention selected according to the predominant baseline abnormality. A donor-cell line displaying excessive H3K9me3 could, for example, undergo carefully titrated modulation of H3K9 methyltransferase activities or another chromatin-opening intervention designed to reduce reprogramming resistance while preserving genomic stability [92]. For a donor-cell line with excessive DNA methylation at relevant developmental regulatory regions, an exploratory and carefully titrated DNMT/TET-directed intervention could be considered as a way to increase epigenetic plasticity without global DNA demethylation. However, DNMT/TET-directed treatment of bovine donor cells has not been sufficiently validated and should thus be regarded as an experimental rather than a routine donor-cell optimization strategy. In contrast, overexpression of TET3 has been directly investigated in bovine reconstructed embryos [24]. Similarly, a donor-cell population displaying pronounced oxidative stress could first undergo metabolic or redox optimization. Combination treatments could subsequently be evaluated, but initial experiments should isolate individual mechanisms and toxicities to distinguish genuine improvements in reprogramming competence from nonspecific cellular stress responses. Following treatment and washout, donor cells should undergo a defined recovery period before Gate A assessment. Release criteria should be based on passage-matched untreated controls cultured under the same conditions and, when available, donor-cell lines with established competence for SCNT. Viability and proliferative recovery should be within predefined acceptable control ranges, and γH2AX positivity, apoptosis, chromosomal abnormalities and mitochondrial dysfunction should not show any biologically relevant increase. The intended epigenetic modification should also be confirmed in the absence of evidence for pervasive epigenetic disruption. Importantly, recovery should be coordinated with donor–oocyte cell-cycle compatibility. Donor cells should be synchronized or sorted in an appropriate G0/G1 phase after the recovery phase and the distribution of the cell cycle should be evaluated immediately before nuclear transfer [52,53]. As no universal validated numerical thresholds for these parameters have been established across bovine donor-cell lines, acceptance limits should be defined in the pilot experiments, taking into consideration assay variability and biological replication, and then evaluated against embryo development and genomic integrity. Nuclear transfer should not be performed using donor cell populations that do not meet these criteria.

Stage 2 begins after fusion and activation. Embryo-stage intervention is selected according to the residual defect detected in SCNT embryos generated from donor-primed cells. Persistent H3K9me3 associated with failure to activate genes within resistant chromatin domains could justify testing transient KDM4-family demethylase delivery [9]. Adequate H3K9me3 remodeling accompanied by persistently low histone acetylation could justify testing a short scriptaid exposure [93]. Similarly, abnormal remethylation at selected developmental regulatory regions could provide a rationale for carefully controlled modulation of DNMT activity. Transient XIST suppression may be tested for the rationale that ectopic XIST expression may be associated with inappropriate repression of X-linked genes. However, the most direct evidence for Xist suppression by RNA interference comes from mouse SCNT embryos [16]. Therefore, the application of this intervention in bovine embryos should be considered experimental and validated in bovine.

Two examples of experimental scenarios may help to clarify the operational basis of the proposed framework. First, a bovine donor-cell line with high H3K9me3 associated chromatin resistance could be subjected to transient, carefully optimized epigenetic priming followed by washout, recovery and assessment of genomic integrity. Reconstructed embryos of these cells would then be tested for persistent H3K9me3 and incomplete activation of embryonic transcriptional programs. If these abnormalities are sustained, transient KDM4E expressions could be investigated as an embryo-stage intervention, supported by bovine evidence that KDM4E-mediated H3K9 demethylation improves EGA and developmental competence [9]. Second, donor-cell priming for chromatin accessibility improvement could be followed by assessment of histone acetylation in reconstructed embryos. If acetylation is still insufficient, short-term treatment with trichostatin A or scriptaid could be attempted as these inhibitors have been shown to increase H4K12 acetylation and improve blastocyst development in bovine SCNT embryos, although the response varied among donor-cell lines [15]. In either case, the potential benefit of sequential treatment is the possibility to overcome residual epigenetic barriers that may not be rectified by donor-cell priming alone, while potentially minimizing unnecessary embryo exposure. However, this advantage is hypothetical and needs to be compared with single-stage interventions.

Importantly, conditional rescue should not be viewed as a real-time molecular screening of single embryos. Since most epigenetic and transcriptomic assays are destructive, treatment selection would initially be based on pilot experiments on parallel cohorts of embryos derived from the same donor-cell line and under similar reconstruction and culture conditions. Representative embryos would be sampled at predetermined developmental stages to identify persistent molecular abnormalities; separate cohorts would be used to assess candidate interventions and developmental outcomes. Molecular assessments to establish a treatment should be carried out in pilot cohorts before the protocol developed is applied to subsequent embryos, particularly when the window for effective intervention is prior to major embryonic genome activation. Having identified the molecular target, timing of treatment and safety profile, the optimized intervention could be prospectively applied to subsequent embryo cohorts without destructive testing of embryos destined for transfer. Thus, conditional rescue constitutes a donor-line- or cohort-level evidence-guided decision rather than an immediate treatment decision for each individual embryo.

The definitive experiment should use a factorial design. At minimum, four groups are required: untreated donor/untreated embryo, primed donor/untreated embryo, untreated donor/treated embryo, and primed donor/treated embryo. This design permits independent evaluation of the effects of donor-cell priming, embryo-stage rescue, and their interaction rather than attributing any improvement to the combined treatment alone [94]. IVF embryos can be an important developmental reference to assess SCNT embryos [17]. Parthenogenetic embryos could also be useful for specific mechanistic comparisons. However, the fact that they contain only maternal genomic contributions and have associated differences in imprinting means that any comparison needs to be interpreted with caution [95]. Evidence for an integrated benefit would require that the combined group demonstrates additive or synergistic molecular and developmental improvement without increased cellular toxicity or developmental abnormalities [45]. Historical controls or comparisons limited to combined treatment versus untreated SCNT embryos cannot resolve these effects.

Molecular endpoints should be prespecified to determine whether an integrated reprogramming strategy corrects the underlying epigenetic defects rather than simply increasing blastocyst yield. Before major EGA, studies should quantify nuclear remodeling events, including H3K9me3 persistence, histone acetylation, DNA methylation and hydroxymethylation dynamics (5mC/5hmC), and DNA damage responses. During the bovine major EGA window, evaluation should focus on activation of embryonic transcriptional programs, suppression of donor-cell transcriptional memory, and restoration of chromatin accessibility at developmentally relevant promoters and enhancers [2,9]. Once the embryo has reached the blastocyst stage, evaluation should not only be based on morphology but also on total cell number, the ratio of inner cell mass to trophectoderm, apoptosis, chromosome integrity, imprinting status and expression of lineage-specific markers [17]. Single-cell RNA sequencing, low-input Assay for Transposase-Accessible Chromatin using sequencing (ATAC-seq), Cleavage Under Targets and Tagmentation (CUT&Tag), and targeted bisulfite sequencing provide powerful approaches for identifying informative biomarkers during protocol development; once validated, however, a smaller targeted molecular panel may be more practical for routine screening [45].

At the molecular scale, persistent accessibility at donor-cell regulatory regions should be differentiated from failure to open embryonic regulatory regions. In bovine SCNT blastocysts, the persistence of donor-cell chromatin accessibility along with H3K4me3 enrichment has been linked to aberrant BCL11B expression and poor inner cell mass proliferation. KDM5B-mediated reduction of H3K4me3 reduced aberrant gene expression and improved ICM proliferation, and BCL11B knockdown added further functional evidence of this pathway [3]. Thus, BCL11B-associated regulatory regions, BCL11B expression and downstream CDKN1A (p21) expression are candidate readouts for persistent somatic memory. Conversely, embryonic reprogramming should be assessed by regaining access and transcription at bovine EGA-linked promoters and enhancers using stage-matched fertilized embryos to find regions that remain closed after SCNT. Candidate loci should be identified from reproducible bovine chromatin-accessibility and transcriptomic data, not inferred from mouse models [86]. In this way, successful intervention would require simultaneous closing of inappropriate donor-derived regulatory regions and opening of embryonic regulatory regions, with appropriate gene expression and improved developmental outcomes. These readouts at the level of the locus require prospective validation prior to routine use.

To distinguish between residual donor-cell memory and normal developmental remodeling, molecular profiles need to be compared between original donor cells, SCNT embryos and stage-matched IVF embryos [3,86]. Persistence of donor-associated regulatory features beyond their expected developmental window, particularly when combined with inappropriate transcription and impaired developmental competence, would support residual somatic memory [3]. Conversely, changes related to normal stage-specific remodeling or altered developmental timing should not be automatically interpreted as reprogramming defects [96].

Gate C addresses a frequent limitation of SCNT studies: improved blastocyst development is often used as the principal measure of success even though preimplantation improvements may not translate into normal post-implantation development [45]. Following embryo transfer, evaluation should therefore include the pregnancy rate, fetal loss, placentome development, fetal growth, gestation length, birth weight, neonatal survival, and clinical assessment [5,8,97]. Placental tissue is particularly informative because abnormalities in H3K27me3-dependent imprinting and extraembryonic lineage development may remain undetected during preimplantation development yet contribute to later developmental failure [6]. Long-term follow-up should include growth, metabolic health, and fertility where feasible.

An integrated strategy also requires detailed reporting of biological materials, experimental conditions, and developmental outcomes to ensure reproducibility. Essential information should include donor-animal identity and cell type, passage number, synchronization procedure, treatment dose, exposure duration and washout and recovery periods; oocyte source and maturation conditions; fusion and activation parameters; embryo culture system; embryo sex when determined; number of biological replicates; and denominators for each developmental endpoint. Epigenetic outcomes should be reported at clearly defined developmental stages and compared with appropriate fertilized embryo controls whenever possible. Such standardized reporting is important because broad descriptions such as “TSA-treated SCNT” can conceal substantial differences in donor-cell state, treatment timing, culture conditions, and biological response, thereby limiting comparison across studies [17].

A practical approach to the assessment of this framework in cattle is conservative donor-cell priming, and embryo-stage intervention only in the presence of a persistent molecular defect. One potential target, supported by bovine SCNT evidence [9], is persistent H3K9me3. Conversely, Xist suppression [16] and inhibition of abnormal DNA remethylation [23] are still experimental in cattle, as the referred intervention studies were performed in mouse SCNT models. Thus, the proposed framework gives priority to targeted stage-specific correction rather than the routine combination of multiple epigenetic modifiers.

## 7. Evaluation Framework, Experimental Design and Translational Endpoints

The translation of epigenetic interventions into cattle production should follow a hierarchy of evidence rather than relying on blastocyst yield alone. The first level is biochemical target engagement, demonstrating that the intervention modifies the intended epigenetic mark at the appropriate developmental stage. The second is transcriptional rescue, demonstrated by appropriately timed activation of embryonic developmental programs and suppression of donor-cell transcriptional memory. The third is cellular competence, demonstrated by normal cleavage, blastocyst development and structure, lineage allocation, chromosome integrity, and apoptosis. The fourth is organismal safety and developmental competence, requiring pregnancy establishment, placental development, birth outcomes, and postnatal health without introducing new developmental abnormalities [45,97]. Table 3 provides a minimum evaluation framework.

The evidence available shows that candidate interventions vary in their readiness for bovine SCNT applications. KDM4E mediated H3K9me3 reduction and short-term HDAC inhibition have direct bovine developmental support and are amenable for further controlled experimental testing [9,15]. The KDM5B-mediated correction of donor-cell transcriptional memory has promising mechanistic evidence in bovines but requires further developmental validation [48]. By contrast, the XIST suppression and the targeted prevention of aberrant DNA remethylation are mainly supported by mouse SCNT studies [16]. The transcription factor-mediated donor-cell priming is poorly validated in cattle [63]. Hence, these approaches must be regarded as experimental strategies rather than established protocols until their developmental efficacy and safety have been demonstrated.

Special attention should be paid to experimental units and replication when designing a SCNT study since embryos derived from the same oocyte collection, donor-cell preparation, or experimental run are not fully independent biological replicates [98]. Binary developmental outcomes, such as cleavage, blastocyst formation, pregnancy establishment and live birth, should preferably be analyzed using generalized linear mixed models with appropriate link functions and random effects for experimental run, donor-cell line, oocyte batch and recipient, where relevant [99]. Treatment effects must be reported with well-defined denominators, effect sizes, confidence intervals and numbers of independent biological replicates. Molecular analysis should also consider clustering and avoid pseudoreplication by treating multiple cells or embryos from the same biological sample as independent observations [100].

Dose response and timing experiments should precede combination testing because epigenetic modifiers often have narrow effective windows. Insufficient exposure may fail to correct the intended molecular barrier, whereas excessive exposure may disturb essential chromatin states, alter developmental gene regulation, or compromise embryo competence [15]. The optimal intervention window should therefore be defined according to each modifier, delivery method, and developmental stage rather than incorporated into a fixed treatment protocol. Drug carryover from treated donor cells should be minimized and, where relevant, experimentally excluded. Similarly, the persistence of delivered mRNA or protein following embryo injection should be quantified where feasible to confirm transient and controlled activity. A combination strategy should not be considered successful if increased blastocyst development is accompanied by increased aneuploidy, DNA damage, imprinting abnormalities, or placental defects [6].

Benchmarking against IVF embryos is necessary but should be interpreted carefully. IVF embryos provide a physiological developmental reference, but this does not imply that every molecular feature of an SCNT embryo must be identical to its IVF counterpart. Differences between SCNT and IVF embryos may reflect altered kinetics of chromatin remodeling and EGA and should not automatically be considered detrimental [96]. Priority should instead be given to molecular abnormalities associated with developmental failure, including persistence of reprogramming-resistant chromatin domains, abnormal timing of embryonic genome activation, imprinting abnormalities, lineage-allocation defects, and impaired placental development [101]. Parthenogenetic embryos can provide useful information about activation-related and maternal cytoplasmic effects but should not be regarded as normal controls for imprinting because they lack a paternal genome and its associated epigenetic contribution [102].

Multi-omics approaches can accelerate biomarker discovery and mechanistic evaluation of reprogramming. Single-cell or low-input transcriptomics can identify heterogeneous reprogramming trajectories and the persistence of donor-cell transcriptional memory in bovine SCNT embryos [37]. ATAC-seq can map accessible regulatory regions, whereas low-input CUT&Tag can characterize histone modifications, including H3K9me3, H3K27me3, and histone acetylation. Bisulfite sequencing or enzymatic methyl-sequencing can provide locus-specific and genome-wide information on DNA methylation states [37]. Chromosome-conformation approaches can further investigate the reorganization of three-dimensional genome architecture during nuclear reprogramming [103]. The integration of these molecular layers may help distinguish embryos that merely reach the blastocyst stage from those displaying more coordinated epigenetic and transcriptional reprogramming [74]. For routine applications, however, a validated targeted biomarker panel is likely to be more practical and cost-effective than comprehensive multi-omics profiling.

Regulatory and animal-welfare considerations favor strategies based on transient exposure, defined washout periods, non-integrating reagents, and comprehensive development and health monitoring because interventions applied during early embryonic development may have long-term consequences. Targeted genome editing of donor cells may provide highly specific manipulation of molecular pathways; however, permanent alteration of epigenetic regulators may have unintended consequences because these factors frequently regulate multiple developmental processes across tissues [104]. Small-molecule epigenetic modifiers are experimentally convenient but often have limited specificity, as many compounds affect multiple chromatin-regulating enzymes and non-target cellular pathways [105]. In contrast, mRNA-, siRNA-, and antisense-based approaches provide an intermediate strategy by allowing transient and, in some cases, sequence-directed modulation without permanent genomic modification [106]. The appropriate platform should therefore be selected according to whether the molecular barrier is global, domain-specific, or locus-specific, and whether transient or sustained modulation is required.

Economic feasibility should be evaluated only after biological efficacy and developmental safety have been established because increased blastocyst production does not necessarily translate into increased production of healthy offspring [107]. An intervention that increases blastocyst yield without improving pregnancy establishment, calf survival, or long-term health may increase recipient-animal use and veterinary costs without providing a practical benefit. Conversely, even a modest increase in live birth efficiency may be valuable when the transferred genotype has exceptional genetic, agricultural, or biomedical importance [1]. Translational evaluation should therefore incorporate both the efficiency of producing healthy offspring by reconstructed oocyte and the efficiency per transferred embryo, together with treatment costs, recipient-animal requirements, pregnancy losses, and neonatal care. Such multidimensional assessment better aligns improvements in SCNT efficiency with responsible livestock application [108].

**Table 3:** Minimum evidence framework for evaluating an integrated bovine SCNT protocol.

Level	Minimum Endpoints	Interpretation	Go/No-Go Criterion	References
Donor-cell release	Viability, proliferative recovery, G0/G1 fraction, karyotype, γH2AX, apoptosis, mitochondrial function, epigenetic target engagement	Confirms reversible priming, genomic integrity, and cell-cycle compatibility	Proceed only when viability, recovery, genomic integrity, and G0/G1 compatibility meet control-based criteria, with confirmed epigenetic target engagement	[50,52]
Early embryo	Fusion/activation, nuclear remodeling, H3K9me3, acetylation, 5mC/5hmC, DNA damage	Determines whether the oocyte is remodeling the primed nucleus appropriately	Reject protocols with target engagement accompanied by DNA/chromosome damage	[9]
Major EGA	Bovine 4–16-cell EGA panel, donor-memory genes, accessibility or transcriptome	Tests conversion from somatic to embryonic regulation	Proceed only when embryonic activation and donor-program shutdown are coordinated	[109]
Blastocyst	Rate, total cells, ICM:TE ratio, apoptosis, ploidy, imprinted and lineage genes	Measures quality rather than morphology alone	Reject gains limited to blastocyst number with reduced cellular quality	[110]
Pregnancy/placenta	Pregnancy establishment, fetal loss, placentomes, vascularization, fetal growth	Detects extraembryonic and imprinting-related failure	Require equal or improved placental and fetal safety	[108]
Postnatal	Birth weight, neonatal survival, clinical chemistry, growth, fertility	Determines genuine cloning success	Adopt only when healthy offspring per oocyte or transfer is improved	[8]

**Abbreviations:** 5hmC, 5-hydroxymethylcytosine; 5mC, 5-methylcytosine; EGA, embryonic genome activation; G0/G1, gap 0/gap 1 phases of the cell cycle; H3K9me3, histone H3 lysine 9 trimethylation; ICM, inner cell mass; SCNT, somatic cell nuclear transfer; TE, trophectoderm; γH2AX, phosphorylated histone H2A.X.

## 8. Challenges and Future Directions

The first challenge is heterogeneity. Donor animals, cell types, passage numbers, oocyte batches, and embryo culture systems differ substantially among SCNT studies, making the development of a universally effective epigenetic treatment unlikely [8]. Future studies should identify predictive biomarkers of donor-cell line response and use baseline molecular defects to guide the selection of appropriate interventions. Machine-learning approaches may eventually integrate donor-cell epigenomic profiles, oocyte-quality parameters, and early embryo imaging features to predict reprogramming competence, although large, standardized multicenter datasets will be required to develop and validate reliable predictive models [1].

The second challenge is specificity. Most currently available epigenetic modifiers act broadly across the genome and may also affect non-histone proteins and other cellular pathways, limiting their ability to correct specific reprogramming defects [8]. CRISPR-based epigenome editing, locus-specific methylation or demethylation and programmable transcriptional regulation are promising experimental approaches to increase targeting specificity [111]. However, their ability to improve epigenetic reprogramming and developmental outcomes in bovine SCNT remains to be established. For exploratory studies, transient delivery of mRNA or ribonucleoprotein complexes may reduce prolonged activity and avoid permanent integration of exogenous DNA [104].

The third challenge is extraembryonic development. Many developmental losses after SCNT are associated with placental abnormalities, yet mechanistic studies have historically focused more extensively on preimplantation embryos than on extraembryonic tissues [108]. Comparative single-cell and spatial profiling of cloned and IVF conceptuses, trophoblast populations, and placental tissues could help identify the earliest molecular abnormalities associated with subsequent developmental failures. H3K27me3-dependent imprinting, X-chromosome regulation, angiogenic signaling, and maternal–conceptus communication represent particularly important areas for investigation because abnormalities in these processes can disrupt fetal and placental development [112]. An embryo-stage intervention that improves EGA but leaves major trophoblast or placental epigenetic abnormalities unresolved may therefore be insufficient to overcome the post-implantation limitations of SCNT.

The fourth challenge is distinguishing causal defects from molecular correlates. Epigenetic abnormality may arise because an embryo is already developmentally compromised rather than being the primary cause of developmental failure. Establishing causality therefore requires perturbation and rescue experiments, together with locus- or domain-specific molecular validation [113]. For example, demonstrating elevated H3K9me3 is insufficient to establish a causal barrier; targeted reduction of H3K9me3 should restore chromatin accessibility and transcription within resistant domains and produce a corresponding improvement in developmental outcomes [9]. Similarly, abnormal DNA methylation should be mechanistically linked to altered transcription and developmental phenotypes rather than interpreted as an isolated biomarker.

The fifth challenge is to determine long-term developmental and health outcomes. In this context, safety means freedom from treatment-related adverse effects after the neonatal period, including abnormal growth, organ dysfunction, metabolic or immune disturbances, reduced fertility, and where relevant, effects in the next generation. Epigenetic alterations may lead to subtle abnormalities that are not detected at birth and require longitudinal follow-up of cloned offspring [108,114].

The sixth challenge is nomenclature and reporting. The term “epigenetic reprogramming” is frequently used broadly to describe changes in DNA methylation or histone modifications, although these modifications differ substantially in genomic distribution, molecular mechanism, developmental timing, and functional consequences [115]. Future studies should specify the molecular mark, genomic location, developmental stage, direction of change, and persistence of the modification. Negative results are also informative because they define ineffective or toxic intervention windows and improve mechanistic understanding. Standardized reporting frameworks and shared datasets would reduce unnecessary duplication, improve reproducibility, and facilitate comparative analyses across studies [116].

In the near term, a rational experimental strategy would be to prioritize conservative combinations supported by mechanistic evidence: a short donor-cell priming step, followed by treatment washout, recovery, and genomic-integrity assessment, and then a transient embryo-stage intervention directed against a demonstrated residual reprogramming barrier. The longer-term objective is precision epigenetic cloning, in which donor-cell line and embryo molecular profiles are used to select the minimum intervention required to promote a coordinated developmental trajectory.

## 9. Conclusions

The low efficiency of bovine SCNT reflects, in substantial part, the difficulty of rapidly converting a differentiated somatic nucleus into an embryonic state capable of supporting normal development. Although interventions targeting either donor cells or reconstructed embryos have improved specific aspects of nuclear reprogramming, these approaches do not fully address the sequential and dynamic nature of the reprogramming process. An integrated strategy could reduce donor cell-derived epigenetic resistance before nuclear transfer and subsequently correct residual defects during early embryonic development.

The framework proposed in this review extends previous dual-stage treatment strategies by connecting donor-cell preparation to molecular assessment and conditional embryo-stage rescue. Its potential advantage lies not in combining multiple epigenetic modifiers per se, but in applying the minimum intervention required to correct a demonstrated stage-specific defect. Importantly, the efficacy and safety of this complete framework have not yet been established in bovine SCNT using factorial experimental designs and developmental endpoints extending beyond blastocyst formation. Placental development and function, pregnancy and live birth outcomes, postnatal health, and reproductive performance will therefore require validation before such an approach can be considered for practical application.

## Figures and Tables

**Table 1:** Major reprogramming barriers in bovine SCNT, candidate stage-specific responses, associated risks and recommended readouts.

Barrier	Evidence	Candidate Response	Key Risk	Readout	References
Persistent H3K9me3	I	Transient KDM4 expression or controlled SUV39H1/2 inhibition	Excessive H3K9 demethylation	H3K9me3; DNA damage	[9,45]
Low histone acetylation	I	Short TSA or scriptaid treatment	Embryo toxicity	H3/H4 acetylation; apoptosis	[15]
Abnormal DNA methylation	I/II	Transient DNMT Knockdown	Imprint loss	5mC/5hmC; imprinted genes	[46,47]
Loss of H3K27me3 imprinting	IV	Targeted locus correction	Uncertain relevance to cattle	Allelic expression; placenta	[6]
Ectopic XIST expression	IV	Transient XIST suppression	Dosage imbalance	XIST; X-linked genes	[16]
Donor transcriptional memory	II	Chromatin correction before EGA	Off-target transcription	Memory genes; EGA markers	[48]
Oxidative and mitochondrial stress	I	Antioxidant support	Excessive ROS suppression	ROS; ATP; apoptosis	[41]

**Note:** Evidence categories: I, direct bovine developmental evidence; II, bovine molecular or mechanistic evidence; III, evidence from closely related livestock species; IV, mechanistic or interventional evidence from mouse, human, or other non-livestock models. Combined categories (I/II) indicate that both developmental and molecular evidence are available. These classifications describe the nature of the supporting evidence and do not necessarily establish the efficacy or safety of the proposed interventions in bovine SCNT. Candidate responses that lack direct bovine intervention evidence should be interpreted as experimental strategies requiring further validation, rather than established protocols for improving bovine SCNT. **Abbreviations:** 5hmC, 5-hydroxymethylcytosine; 5mC, 5-methylcytosine; ATP, adenosine triphosphate; DNMT, DNA methyltransferase; EGA, embryonic genome activation; H3/H4, histones H3 and H4; H3K27me3, histone H3 lysine 27 trimethylation; H3K9me3, histone H3 lysine 9 trimethylation; KDM4, lysine demethylase 4 family; ROS, reactive oxygen species; SCNT, somatic cell nuclear transfer; SUV39H1/2, suppressor of variegation 3–9 homologs 1 and 2; TSA, trichostatin A; XIST, X-inactive specific transcript.

## Data Availability

No new datasets were generated or analyzed in this review.

## References

[1] Galli C., Lazzari G. (2021). 25th ANNIVERSARY OF CLONING BY SOMATIC-CELL NUCLEAR TRANSFER: Current applications of SCNT in advanced breeding and genome editing in livestock. Reproduction.

[2] Matoba S., Liu Y., Lu F., Iwabuchi K.A., Shen L., Inoue A., Zhang Y. (2014). Embryonic development following somatic cell nuclear transfer impeded by persisting histone methylation. Cell.

[3] Huang Y., Zhang J., Li X., Wu Z., Xie G., Wang Y., Liu Z., Jiao M., Zhang H., Shi B. (2023). Chromatin accessibility memory of donor cells disrupts bovine somatic cell nuclear transfer blastocysts development. FASEB J. Off. Publ. Fed. Am. Soc. Exp. Biol..

[4] Inoue K., Ogonuki N., Kamimura S., Inoue H., Matoba S., Hirose M., Honda A., Miura K., Hada M., Hasegawa A. (2020). Loss of H3K27me3 imprinting in the *Sfmbt2* miRNA cluster causes enlargement of cloned mouse placentas. Nat. Commun..

[5] Heyman Y., Chavatte-Palmer P., LeBourhis D., Camous S., Vignon X., Renard J.P. (2002). Frequency and occurrence of late-gestation losses from cattle cloned embryos. Biol. Reprod..

[6] Matoba S., Wang H., Jiang L., Lu F., Iwabuchi K.A., Wu X., Inoue K., Yang L., Press W., Lee J.T. (2018). Loss of H3K27me3 Imprinting in Somatic Cell Nuclear Transfer Embryos Disrupts Post-Implantation Development. Cell Stem Cell.

[7] Zhang X., Gao S., Liu X. (2021). Advance in the Role of Epigenetic Reprogramming in Somatic Cell Nuclear Transfer-Mediated Embryonic Development. Stem Cells Int..

[8] Panarace M., Agüero J.I., Garrote M., Jauregui G., Segovia A., Cané L., Gutiérrez J., Marfil M., Rigali F., Pugliese M. (2007). How healthy are clones and their progeny: 5 years of field experience. Theriogenology.

[9] Liu X., Wang Y., Gao Y., Su J., Zhang J., Xing X., Zhou C., Yao K., An Q., Zhang Y. (2018). H3K9 demethylase KDM4E is an epigenetic regulator for bovine embryonic development and a defective factor for nuclear reprogramming. Development.

[10] Chung Y.G., Matoba S., Liu Y., Eum J.H., Lu F., Jiang W., Lee J.E., Sepilian V., Cha K.Y., Lee D.R. (2015). Histone Demethylase Expression Enhances Human Somatic Cell Nuclear Transfer Efficiency and Promotes Derivation of Pluripotent Stem Cells. Cell Stem Cell.

[11] Xu R., Zhu Q., Zhao Y., Chen M., Yang L., Shen S., Yang G., Shi Z., Zhang X., Shi Q. (2023). Unreprogrammed H3K9me3 prevents minor zygotic genome activation and lineage commitment in SCNT embryos. Nat. Commun..

[12] Ming H., Sun J., Pasquariello R., Gatenby L., Herrick J.R., Yuan Y., Pinto C.R., Bondioli K.R., Krisher R.L., Jiang Z. (2021). The landscape of accessible chromatin in bovine oocytes and early embryos. Epigenetics.

[13] Saini M., Selokar N.L., Revey T., Singla S.K., Chauhan M.S., Palta P., Madan P. (2014). Trichostatin A alters the expression of cell cycle controlling genes and microRNAs in donor cells and subsequently improves the yield and quality of cloned bovine embryos in vitro. Theriogenology.

[14] Wang Y.S., Xiong X.R., An Z.X., Wang L.J., Liu J., Quan F.S., Hua S., Zhang Y. (2011). Production of cloned calves by combination treatment of both donor cells and early cloned embryos with 5-aza-2/-deoxycytidine and trichostatin A. Theriogenology.

[15] Akagi S., Matsukawa K., Mizutani E., Fukunari K., Kaneda M., Watanabe S., Takahashi S. (2011). Treatment with a histone deacetylase inhibitor after nuclear transfer improves the preimplantation development of cloned bovine embryos. J. Reprod. Dev..

[16] Matoba S., Inoue K., Kohda T., Sugimoto M., Mizutani E., Ogonuki N., Nakamura T., Abe K., Nakano T., Ishino F. (2011). RNAi-mediated knockdown of *Xist* can rescue the impaired postimplantation development of cloned mouse embryos. Proc. Natl. Acad. Sci. USA.

[17] Matoba S., Zhang Y. (2018). Somatic Cell Nuclear Transfer Reprogramming: Mechanisms and Applications. Cell Stem Cell.

[18] Zhou C.Y., Heald R. (2023). Principles of genome activation in the early embryo. Curr. Opin. Genet. Dev..

[19] Liu B., Yan J., Li J., Xia W. (2023). The role of BDNF, YBX1, CENPF, ZSCAN4, TEAD4, GLIS1 and USF1 in the activation of the embryonic genome in bovine embryos. Int. J. Mol. Sci..

[20] Allshire R.C., Madhani H.D. (2018). Ten principles of heterochromatin formation and function. Nat. Rev. Mol. Cell Biol..

[21] Janssen A., Colmenares S.U., Karpen G.H. (2018). Heterochromatin: Guardian of the Genome. Annu. Rev. Cell Dev. Biol..

[22] Smith Z.D., Meissner A. (2013). DNA methylation: Roles in mammalian development. Nat. Rev. Genet..

[23] Gao R., Wang C., Gao Y., Xiu W., Chen J., Kou X., Zhao Y., Liao Y., Bai D., Qiao Z. (2018). Inhibition of Aberrant DNA Re-methylation Improves Post-implantation Development of Somatic Cell Nuclear Transfer Embryos. Cell Stem Cell.

[24] Zhang J., Hao L., Wei Q., Zhang S., Cheng H., Zhai Y., Jiang Y., An X., Li Z., Zhang X. (2020). *TET3* overexpression facilitates DNA reprogramming and early development of bovine SCNT embryos. Reproduction.

[25] Pappalardo X.G., Barra V. (2021). Losing DNA methylation at repetitive elements and breaking bad. Epigenet. Chromatin.

[26] Kishigami S., Mizutani E., Ohta H., Hikichi T., Thuan N.V., Wakayama S., Bui H.T., Wakayama T. (2006). Significant improvement of mouse cloning technique by treatment with trichostatin A after somatic nuclear transfer. Biochem. Biophys. Res. Commun..

[27] Zhang Y., Li J., Villemoes K., Pedersen A.M., Purup S., Vajta G. (2007). An epigenetic modifier results in improved in vitro blastocyst production after somatic cell nuclear transfer. Cloning Stem Cells.

[28] Van Thuan N., Bui H.-T., Kim J.-H., Hikichi T., Wakayama S., Kishigami S., Mizutani E., Wakayama T. (2009). The histone deacetylase inhibitor scriptaid enhances nascent mRNA production and rescues full-term development in cloned inbred mice. Reproduction.

[29] Miyamoto K., Tsukiyama T., Yang Y., Li N., Minami N., Yamada M., Imai H. (2009). Cell-free extracts from mammalian oocytes partially induce nuclear reprogramming in somatic cells. Biol. Reprod..

[30] Göttlicher M., Minucci S., Zhu P., Krämer O.H., Schimpf A., Giavara S., Sleeman J.P., Lo Coco F., Nervi C., Pelicci P.G. (2001). Valproic acid defines a novel class of HDAC inhibitors inducing differentiation of transformed cells. EMBO J..

[31] Meng J., Zhang H.-H., Zhou C., Li C., Zhang F., Mei Q. (2012). The histone deacetylase inhibitor trichostatin A induces cell cycle arrest and apoptosis in colorectal cancer cells via p53-dependent and -independent pathways. Oncol. Rep..

[32] Inoue A., Jiang L., Lu F., Suzuki T., Zhang Y. (2017). Maternal H3K27me3 controls DNA methylation-independent imprinting. Nature.

[33] Li Y., Sun S., Xu Y., Zhang J., Du Y., Cao Y., Liao Z., Xie Y., Bian X., Huang J. (2025). Efficient Somatic Cell Nuclear Transfer by Overcoming Both Pre- and Post-Implantation Epigenetic Barriers. Adv. Sci..

[34] Gendrel A.-V., Heard E. (2014). Noncoding RNAs and epigenetic mechanisms during X-chromosome inactivation. Annu. Rev. Cell Dev. Biol..

[35] Su J.M., Yang B., Wang Y.S., Li Y.Y., Xiong X.R., Wang L.J., Guo Z.K., Zhang Y. (2011). Expression and methylation status of imprinted genes in placentas of deceased and live cloned transgenic calves. Theriogenology.

[36] Asami M., Lam B.Y.H., Ma M.K., Rainbow K., Braun S., VerMilyea M.D., Yeo G.S.H., Perry A.C.F. (2022). Human embryonic genome activation initiates at the one-cell stage. Cell Stem Cell.

[37] Zhao L., Long C., Zhao G., Su J., Ren J., Sun W., Wang Z., Zhang J., Liu M., Hao C. (2022). Reprogramming barriers in bovine cells nuclear transfer revealed by single-cell RNA-seq analysis. J. Cell. Mol. Med..

[38] Bosnakovski D., Gearhart M.D., Ho Choi S., Kyba M. (2021). Dux facilitates post-implantation development, but is not essential for zygotic genome activation. Biol. Reprod..

[39] Halstead M.M., Ma X., Zhou C., Schultz R.M., Ross P.J. (2020). Chromatin remodeling in bovine embryos indicates species-specific regulation of genome activation. Nat. Commun..

[40] Li W., Liu Y., Wang L., Dilixiati A., Cheng Z., Chang Z., Ma X., He Z., Liang Y., Wang Y. (2025). Heterogeneity in somatic cell nuclear transfer embryo reprogramming across mammalian species. Biol. Reprod..

[41] Bae H.K., Hwang I.S., Kim J.Y., Lee S.Y., Park C.K., Yang B.K., Cheong H.T. (2015). Antioxidant treatment during manipulation procedures prevents mitochondrial and DNA damage and enhances nuclear reprogramming of bovine somatic cell nuclear transfer embryos. Reprod. Fertil. Dev..

[42] Carey B.W., Finley L.W., Cross J.R., Allis C.D., Thompson C.B. (2015). Intracellular α-ketoglutarate maintains the pluripotency of embryonic stem cells. Nature.

[43] Hwang I.S., Bae H.K., Cheong H.T. (2013). Mitochondrial and DNA damage in bovine somatic cell nuclear transfer embryos. J. Vet. Sci..

[44] Blaschke K., Ebata K.T., Karimi M.M., Zepeda-Martínez J.A., Goyal P., Mahapatra S., Tam A., Laird D.J., Hirst M., Rao A. (2013). Vitamin C induces Tet-dependent DNA demethylation and a blastocyst-like state in ES cells. Nature.

[45] Meng F., Stamms K., Bennewitz R., Green A., Oback F., Turner P., Wei J., Oback B. (2020). Targeted histone demethylation improves somatic cell reprogramming into cloned blastocysts but not postimplantation bovine concepti. Biol. Reprod..

[46] Wang L., Liu L., Wang Y., Li N., Zhu H., Chen M., Bai J., Pang Y., Zhang Y., Zhang H. (2021). Aberrant Epigenetic Reprogramming in the First Cell Cycle of Bovine Somatic Cell Nuclear Transfer Embryos. Cell. Reprogram..

[47] Jafari S., Hosseini M.S., Hajian M., Forouzanfar M., Jafarpour F., Abedi P., Ostadhosseini S., Abbasi H., Gourabi H., Shahverdi A.H. (2011). Improved in vitro development of cloned bovine embryos using S-adenosylhomocysteine, a non-toxic epigenetic modifying reagent. Mol. Reprod. Dev..

[48] Zhou C., Zhang J., Zhang M., Wang D., Ma Y., Wang Y., Wang Y., Huang Y., Zhang Y. (2020). Transcriptional memory inherited from donor cells is a developmental defect of bovine cloned embryos. FASEB J. Off. Publ. Fed. Am. Soc. Exp. Biol..

[49] Liu J., Wang Y., Su J., Luo Y., Quan F., Zhang Y. (2013). Nuclear donor cell lines considerably influence cloning efficiency and the incidence of large offspring syndrome in bovine somatic cell nuclear transfer. Reprod. Domest. Anim. Zuchthyg..

[50] Poehland R., Al-Rostum F., Becker F., Viergutz T., Brunner R.M., Kanitz W., Bhojwani S. (2007). Donor cell lines considerably affect the outcome of somatic nuclear transfer in the case of bovines. J. Reprod. Dev..

[51] Setiawan H., Irawan A. (2026). Influence of different cell passage numbers on bovine cloned embryo: A systematic review and meta-analysis. Reprod. Biol..

[52] Nguyen V.K., Somfai T., Salamone D., Thu Huong V.T., Le Thi Nguyen H., Huu Q.X., Hoang A.T., Phan H.T., Thi Pham Y.K., Pham L.D. (2021). Optimization of donor cell cycle synchrony, maturation media and embryo culture system for somatic cell nuclear transfer in the critically endangered Vietnamese Ỉ pig. Theriogenology.

[53] Rodrigues L.L.V., Moura Y.B.F., Viana J., de Oliveira L.R.M., Praxedes É.A., Vieira J.B., Sales S.L.A., Silva H.V.R., Luciano M., Pessoa C. (2023). Full confluency, serum starvation, and roscovitine for inducing arrest in the G_0_/G_1_ phase of the cell cycle in puma skin-derived fibroblast lines. Anim. Reprod..

[54] Zhang Y., Qu P., Ma X., Qiao F., Ma Y., Qing S., Zhang Y., Wang Y., Cui W. (2018). Tauroursodeoxycholic acid (TUDCA) alleviates endoplasmic reticulum stress of nuclear donor cells under serum starvation. PLoS ONE.

[55] Secchia S., Beilinson V., Chen X., Gucwa M., Denson L.A., Miraldi E.R., Weirauch M.T., Ikegami K. (2025). Starvation activates ECM-remodeling gene transcription and putative enhancers in fibroblasts despite inducing quiescence. Cell Rep..

[56] Lee M.-J., Kim S.-W., Lee H.-G., Im G., Yang B., Kim N.-H., Kim D.H. (2011). Trichostatin A promotes the development of bovine somatic cell nuclear transfer embryos. J. Reprod. Dev..

[57] Zeller P., Padeken J., van Schendel R., Kalck V., Tijsterman M., Gasser S.M. (2016). Histone H3K9 methylation is dispensable for *Caenorhabditis elegans* development but suppresses RNA:DNA hybrid-associated repeat instability. Nat. Genet..

[58] Orta M.L., Calderón-Montaño J.M., Domínguez I., Pastor N., Burgos-Morón E., López-Lázaro M., Cortés F., Mateos S., Helleday T. (2013). 5-Aza-2′-deoxycytidine causes replication lesions that require Fanconi anemia-dependent homologous recombination for repair. Nucleic Acids Res..

[59] Gill D., Parry A., Santos F., Okkenhaug H., Todd C.D., Hernando-Herraez I., Stubbs T.M., Milagre I., Reik W. (2022). Multi-omic rejuvenation of human cells by maturation phase transient reprogramming. eLife.

[60] Kashyap V., Rezende N.C., Scotland K.B., Shaffer S.M., Persson J.L., Gudas L.J., Mongan N.P. (2009). Regulation of stem cell pluripotency and differentiation involves a mutual regulatory circuit of the NANOG, OCT4, and SOX2 pluripotency transcription factors with polycomb repressive complexes and stem cell microRNAs. Stem Cells Dev..

[61] Lu J.Y., Tu W.B., Li R., Weng M., Sanketi B.D., Yuan B., Reddy P., Rodriguez Esteban C., Izpisua Belmonte J.C. (2025). Prevalent mesenchymal drift in aging and disease is reversed by partial reprogramming. Cell.

[62] Chondronasiou D., Martínez de Villarreal J., Melendez E., Lynch C.J., Pozo N.D., Kovatcheva M., Aguilera M., Prats N., Real F.X., Serrano M. (2022). Deciphering the roadmap of in vivo reprogramming toward pluripotency. Stem Cell Rep..

[63] Machado L.S., Borges C.M., de Lima M.A., Sangalli J.R., Therrien J., Pessôa L.V.F., Fantinato Neto P., Perecin F., Smith L.C., Meirelles F.V. (2023). Exogenous OCT4 and SOX2 Contribution to In Vitro Reprogramming in Cattle. Biomedicines.

[64] Powell A.M., Talbot N.C., Wells K.D., Kerr D.E., Pursel V.G., Wall R.J. (2004). Cell donor influences success of producing cattle by somatic cell nuclear transfer. Biol. Reprod..

[65] Akagi S., Matsukawa K., Takahashi S. (2014). Factors affecting the development of somatic cell nuclear transfer embryos in Cattle. J. Reprod. Dev..

[66] Feng Y., Zhao X., Li Z., Luo C., Ruan Z., Xu J., Shen P., Deng Y., Jiang J., Shi D. (2021). Histone Demethylase KDM4D Could Improve the Developmental Competence of Buffalo (*Bubalus bubalis)* Somatic Cell Nuclear Transfer (SCNT) Embryos. Microsc. Microanal..

[67] Onosetale D., Haider Z., Perera C.D., Ullah S., Lee S.H., Lee S.E., Kim J., Lee M.J., Idrees M., Kong I.K. (2027). Epigenetic reduction OF H3K9me3 and H3K27me3 by RK-701 and GSK 126 improves the developmental competence of bovine SCNT embryos. Theriogenology.

[68] Kamimura S., Inoue K., Mizutani E., Kim J.M., Inoue H., Ogonuki N., Miyamoto K., Ihashi S., Itami N., Wakayama T. (2021). Improved development of mouse somatic cell nuclear transfer embryos by chlamydocin analogues, class I and IIa histone deacetylase inhibitors. Biol. Reprod..

[69] Zhe X., Zhang L., Ding R., Ma H., Zhang Y., Hao F., Liu D., Li Y. (2025). Chemical reprogramming regulates Tip60 expression to improve cleavage rates in somatic cell nuclear transfer reconstituted embryos of cashmere goats. Front. Vet. Sci..

[70] Akagi S., Matsukawa K. (2022). Effects of Trichostatin A on the Timing of the First Cleavage and In Vitro Developmental Potential of Bovine Somatic Cell Nuclear Transfer Embryos. Cell. Reprogram..

[71] Gao S., Wang Z., Ma J., Wang J., Xing X., Shen C., Niu Z., Li H., Zhang S., Zhang K. (2022). Effect of ACY-1215 on cytoskeletal remodeling and histone acetylation in bovine somatic cell nuclear transfer embryos. Theriogenology.

[72] Dang Y., Li S., Zhao P., Xiao L., Wang L., Shi Y., Luo L., Wang S., Wang H., Zhang K. (2022). The lysine deacetylase activity of histone deacetylases 1 and 2 is required to safeguard zygotic genome activation in mice and cattle. Development.

[73] Ma P., Schultz R.M. (2008). Histone deacetylase 1 (HDAC1) regulates histone acetylation, development, and gene expression in preimplantation mouse embryos. Dev. Biol..

[74] Xu R., Li Y., Wu Y., Liu X., Gao S. (2025). Epigenetic regulation in early embryo development: From zygotic genome activation to the first lineage specification. Trends Genet..

[75] Hackett J.A., Surani M.A. (2013). DNA methylation dynamics during the mammalian life cycle. Philos. Trans. R. Soc. Lond. Ser. B Biol. Sci..

[76] Yu T., Meng R., Song W., Sun H., An Q., Zhang C., Zhang Y., Su J. (2023). ZFP57 regulates DNA methylation of imprinted genes to facilitate embryonic development of somatic cell nuclear transfer embryos in Holstein cows. J. Dairy. Sci..

[77] Stäubli A., Peters A. (2021). Mechanisms of maternal intergenerational epigenetic inheritance. Curr. Opin. Genet. Dev..

[78] Wang L.-Y., Li Z.-K., Wang L.-B., Liu C., Sun X.-H., Feng G.-H., Wang J.-Q., Li Y.-F., Qiao L.-Y., Nie H. (2020). Overcoming Intrinsic H3K27me3 Imprinting Barriers Improves Post-implantation Development after Somatic Cell Nuclear Transfer. Cell Stem Cell.

[79] Riso V., Cammisa M., Kukreja H., Anvar Z., Verde G., Sparago A., Acurzio B., Lad S., Lonardo E., Sankar A. (2016). ZFP57 maintains the parent-of-origin-specific expression of the imprinted genes and differentially affects non-imprinted targets in mouse embryonic stem cells. Nucleic Acids Res..

[80] Borensztein M., Syx L., Ancelin K., Diabangouaya P., Picard C., Liu T., Liang J.B., Vassilev I., Galupa R., Servant N. (2017). Xist-dependent imprinted X inactivation and the early developmental consequences of its failure. Nat. Struct. Mol. Biol..

[81] Nakamura M., Ivec A., Gao Y., Qi L. (2021). Durable CRISPR-Based Epigenetic Silencing. Biodesign Res..

[82] Deng X., Berletch J.B., Nguyen D.K., Disteche C.M. (2014). X chromosome regulation: Diverse patterns in development, tissues and disease. Nat. Rev. Genet..

[83] Heard E., Disteche C.M. (2006). Dosage compensation in mammals: Fine-tuning the expression of the X chromosome. Genes. Dev..

[84] Itoh Y., Arnold A. (2014). X chromosome regulation of autosomal gene expression in bovine blastocysts. Chromosoma.

[85] Yu B., van Tol H.T.A., Stout T.A.E., Roelen B.A.J. (2020). Initiation of X Chromosome Inactivation during Bovine Embryo Development. Cells.

[86] Graf A., Krebs S., Zakhartchenko V., Schwalb B., Blum H., Wolf E. (2014). Fine mapping of genome activation in bovine embryos by RNA sequencing. Proc. Natl. Acad. Sci. USA.

[87] Takahashi K., Ross P.J., Sawai K. (2019). The necessity of ZSCAN4 for preimplantation development and gene expression of bovine embryos. J. Reprod. Dev..

[88] Su J., Wang Y., Xing X., Zhang L., Sun H., Zhang Y. (2015). Melatonin significantly improves the developmental competence of bovine somatic cell nuclear transfer embryos. J. Pineal Res..

[89] Malpotra S., Goel P., Shyam S., Singh M.K., Palta P. (2022). Global DNA methylation profiles of buffalo (*Bubalus bubalis*) preimplantation embryos produced by handmade cloning and in vitro fertilization. Sci. Rep..

[90] Halsall J., Andrews S., Krueger F., Rutledge C., Ficz G., Reik W., Turner B. (2021). Histone modifications form a cell-type-specific chromosomal bar code that persists through the cell cycle. Sci. Rep..

[91] Lavagi I., Krebs S., Simmet K., Beck A., Zakhartchenko V., Wolf E., Blum H. (2018). Single-cell RNA sequencing reveals developmental heterogeneity of blastomeres during major genome activation in bovine embryos. Sci. Rep..

[92] Srirattana K., Kaneda M., Parnpai R. (2022). Strategies to Improve the Efficiency of Somatic Cell Nuclear Transfer. Int. J. Mol. Sci..

[93] Iager A.E., Ragina N.P., Ross P.J., Beyhan Z., Cunniff K., Rodriguez R.M., Cibelli J.B. (2008). Trichostatin A improves histone acetylation in bovine somatic cell nuclear transfer early embryos. Cloning Stem Cells.

[94] Montgomery D.C. (2017). Design and Analysis of Experiments.

[95] Kono T., Obata Y., Wu Q., Niwa K., Ono Y., Yamamoto Y., Park E.S., Seo J.S., Ogawa H. (2004). Birth of parthenogenetic mice that can develop to adulthood. Nature.

[96] Cuthbert J.M., Russell S.J., Polejaeva I.A., Meng Q., White K.L., Benninghoff A.D. (2021). Comparing mRNA and sncRNA profiles during the maternal-to-embryonic transition in bovine IVF and scNT embryos. Biol. Reprod..

[97] Hill J.R., Burghardt R.C., Jones K., Long C.R., Looney C.R., Shin T., Spencer T.E., Thompson J.A., Winger Q.A., Westhusin M.E. (2000). Evidence for placental abnormality as the major cause of mortality in first-trimester somatic cell cloned bovine fetuses. Biol. Reprod..

[98] Aarts E., Verhage M., Veenvliet J.V., Dolan C.V., van der Sluis S. (2014). A solution to dependency: Using multilevel analysis to accommodate nested data. Nat. Neurosci..

[99] Bolker B.M., Brooks M.E., Clark C.J., Geange S.W., Poulsen J.R., Stevens M.H., White J.S. (2009). Generalized linear mixed models: A practical guide for ecology and evolution. Trends Ecol. Evol..

[100] Lazic S.E. (2010). The problem of pseudoreplication in neuroscientific studies: Is it affecting your analysis?. BMC Neurosci..

[101] de Macedo M.P., Glanzner W.G., Gutierrez K., Bordignon V. (2021). Chromatin role in early programming of embryos. Anim. Front..

[102] Kaneda M., Takahashi M., Yamanaka K., Saito K., Taniguchi M., Akagi S., Watanabe S., Nagai T. (2017). Epigenetic analysis of bovine parthenogenetic embryonic fibroblasts. J. Reprod. Dev..

[103] Stevens T.J., Lando D., Basu S., Atkinson L.P., Cao Y., Lee S.F., Leeb M., Wohlfahrt K.J., Boucher W., O’Shaughnessy-Kirwan A. (2017). 3D structures of individual mammalian genomes studied by single-cell Hi-C. Nature.

[104] Anzalone A.V., Koblan L.W., Liu D.R. (2020). Genome editing with CRISPR–Cas nucleases, base editors, transposases and prime editors. Nat. Biotechnol..

[105] Timmers H.T.M., Tora L. (2018). Transcript Buffering: A Balancing Act between mRNA Synthesis and mRNA Degradation. Mol. Cell.

[106] Kulkarni J.A., Cullis P.R., Van Der Meel R. (2018). Lipid nanoparticles enabling gene therapies: From concepts to clinical utility. Nucleic Acid. Ther..

[107] Aherin D.G., Bormann J.M., Heier Stamm J.L., MacNeil M.D., Weaber R.L. (2018). Decision-making tools: Stochastic simulation model accounting for the impacts of biological variation on success of bovine embryo transfer programs. Transl. Anim. Sci..

[108] Chavatte-Palmer P., Camous S., Jammes H., Le Cleac’h N., Guillomot M., Lee R.S. (2012). Review: Placental perturbations induce the developmental abnormalities often observed in bovine somatic cell nuclear transfer. Placenta.

[109] Grow E.J., Liu Y., Fan Z., Perisse I.V., Patrick T., Regouski M., Shadle S., Polejaeva I., White K.L., Cairns B.R. (2023). Chromatin Reprogramming of In Vitro Fertilized and Somatic Cell Nuclear Transfer Bovine Embryos During Embryonic Genome Activation. bioRxiv.

[110] Akagi S., Geshi M., Nagai T. (2013). Recent progress in bovine somatic cell nuclear transfer. Anim. Sci. J. Nihon Chikusan Gakkaiho.

[111] Kwon D.Y., Zhao Y.-T., Lamonica J.M., Zhou Z. (2017). Locus-specific histone deacetylation using a synthetic CRISPR-Cas9-based HDAC. Nat. Commun..

[112] Kramer A., Jansson T., Bale T., Powell T. (2023). Maternal-fetal cross-talk via the placenta: Influence on offspring development and metabolism. Development.

[113] Hanna C., Kelsey G. (2021). Features and mechanisms of canonical and noncanonical genomic imprinting. Genes Dev..

[114] Keefer C.L. (2015). Artificial cloning of domestic animals. Proc. Natl. Acad. Sci. USA.

[115] Allis C.D., Jenuwein T. (2016). The molecular hallmarks of epigenetic control. Nat. Rev. Genet..

[116] Percie du Sert N., Hurst V., Ahluwalia A., Alam S., Avey M.T., Baker M., Browne W.J., Clark A., Cuthill I.C., Dirnagl U. (2020). The ARRIVE guidelines 2.0: Updated guidelines for reporting animal research. Exp. Physiol..

